# Unraveling the Lipidome and Antioxidant Activity of Native *Bifurcaria bifurcata* and Invasive *Sargassum muticum* Seaweeds: A Lipid Perspective on How Systemic Intrusion May Present an Opportunity

**DOI:** 10.3390/antiox9070642

**Published:** 2020-07-21

**Authors:** Fábio Santos, João P. Monteiro, Daniela Duarte, Tânia Melo, Diana Lopes, Elisabete da Costa, Maria Rosário Domingues

**Affiliations:** 1 Mass Spectrometry Centre, LAQV-REQUIMTE, Department of Chemistry, University of Aveiro, Santiago University Campus, 3810-193 Aveiro, Portugal; fabiofs379@gmail.com (F.S.); jpspmonteiro@yahoo.com (J.P.M.); danieladuarte98@ua.pt (D.D.); taniamelo@ua.pt (T.M.); dianasalzedaslopes@ua.pt (D.L.); elisabetecosta@ua.pt (E.d.C.); 2 CESAM—Centre for Environmental and Marine Studies, Department of Chemistry, University of Aveiro, Santiago University Campus, 3810-193 Aveiro, Portugal

**Keywords:** bioactivity, lipidomics, mass spectrometry, nutritional quality, polyunsaturated fatty acids, macroalgae, antioxidant

## Abstract

Brown seaweeds are known to present components with appealing bioactive properties eliciting great interest for industrial applications. However, their lipid content is generally disregarded beyond their fatty acid (FA) composition. This study thoroughly characterized the lipid profile of two brown seaweeds collected from Portuguese coast, the native *Bifurcaria bifurcata* and the invasive *Sargassum muticum* species, and bioprospecting for antioxidant activity. An integrated state-of-the-art approach including gas chromatography-mass spectrometry (GC–MS) and liquid chromatography-mass spectrometry (HILIC–ESI-MS/MS), allowed a comprehensive picture of FA and polar lipid content. Polar lipid profile of *B. bifurcata* and *S. muticum* included 143 and 217 lipid species respectively, distributed between glycolipids, phospholipids, and betaine lipids. Some of the lipid species found have been assigned biological activity and contain of *n*-3 and *n*-6 FA. *Sargassum muticum* presented the highest *n*-3 FA content. Low concentrations of extracts of both seaweeds displayed antioxidant activity, with *S. muticum* presenting more promising results. These findings contribute to the nutritional and industrial exploitation of both seaweeds, highlighting their relevance as viable sources of bioactive and added-value compounds. *Sargassum muticum* presented interesting lipid composition and bioactivity, which may represent an accessible opportunity for the exploitation of this invasive seaweed, especially taking advantage of *Sargassum* blooms.

## 1. Introduction

Over the last decades consumer priority turned its focus to lifestyle, healthiness, and well-being, without neglecting environmental and sustainability concerns [1]. These concerns, along with the increasing demand for natural compounds and functional foods, justified a new look at the composition of seaweeds, since it is well recognized that these marine resources are a natural and sustainable source of natural compounds [2,3]. This new paradigm led to an increasing interest in seaweed utilization for food, cosmetic, agricultural, pharmaceutical, biomedical, and nutraceutical applications [4,5]. The Portuguese coast hosts 1909 different marine algae, 243 of which belong to Phaeophyceae group [6], where native seaweeds like *Bifurcaria bifurcata* (R. Ross, 1958) and invasive species like *Sargassum muticum* ((Yendo) Fensholt, 1955) share the same habitats. *Bifurcaria bifurcata* inhabits Atlantic Ocean and can be found from southern limit of Morocco to north-western Ireland [7,8]. As for *S. muticum*, from the shorelines of Gulf of Mexico in the Atlantic Ocean, it was pushed by maritime currents into the North Atlantic, composing the so-called Sargasso Sea [9]. In 2018 the total amount of *S. muticum* biomass was estimated to attain a whopping amount of 20 million tones, impacting activities like sailing or fishing and causing great environmental disturbances in beaches and reef lagoons [9,10]. *Sargassum muticum* invasion into new territories brought subsequent changes to local native species, causing the decrease in the occurrence of some native perennial seaweeds [11,12], or even leading to the loss of native genotypes [13,14]. Moreover, it has been suggested that invasive seaweeds have the potential to change epifaunal communities, and therefore alter the dynamics of entire ecosystems [15]. Until now there is no permanent method to remove this invasive species, nevertheless several efforts to fully take advantage of this biomass have been proposed [16,17]. In fact, invasive seaweeds such as *S. muticum* are potentially interesting and a viable natural resource for a global marine-derived drugs market, predicted to represent $21,955.6 billion by 2025 [18].

Brown seaweeds have been studied mostly due to the intrinsic properties of their polysaccharides, which are largely used by the hydrocolloid industry [19,20], but they also have been explored in the cosmetic and textile industries, and probed for biomedical and pharmaceutical applications [17,21]. Other compounds such as proteins, minerals, pigments, polyphenols, and polyunsaturated fatty acids (PUFA) justified the interest for distinct applications [22,23]. The total lipid content in brown seaweeds accounts for ca. 8% of dry weigh (DW) biomass and is commonly characterized in terms of fatty acid composition and recognized for its high content in PUFA. Lipid fractions from brown seaweeds, such as *B. bifurcata* and *S. muticum*, include an interesting content in PUFA eicosapentaenoic acid (20:5 *n*-3), docosahexaenoic acid (22:6 *n*-3), octadecatetraenoic acid (18:4 *n*-3), α-linolenic acid (18:3 *n*-3), and eicosatetraenoic acid (20:4 *n*-6) [7,24,25,26,27]. This is especially important because *n*-3 PUFA are claimed to be beneficial for the prevention of cardiovascular diseases and other chronic diseases [21,28,29].

Antioxidant effects are among the most searched bioactivities for natural products, including lipids [30,31]. Natural antioxidant products, with radical scavenging activity and capacity to neutralize reactive oxygen species (ROS) protect cells from ROS-induced cellular damage [30,31,32,33], thereby reducing the risk of diseases associated with oxidative stress [34,35]. Seaweed antioxidants are sustainable alternatives to synthetic antioxidants, in line with consumer preference towards natural substances, and can be use as functional ingredients in food or in cosmetics products [25]. In general, in vitro studies have been mainly devoted to the survey of lipophilic compounds from seaweeds such as carotenoids, some polyphenols, and flavonoids exhibiting antioxidant activity [21,31]. Particularly, *B. bifurcata* dichloromethane extract, mainly composed of diterpenes, fatty acids (FA), and sterols, showed antioxidant, anti-inflammatory, and antibacterial activities [24] and provided interesting information for the further exploitation of this seaweed. Moreover, active components of the ethyl acetate, ethanol, and methanol extracts of *S. serratifolium* showed radical scavenging activities [31], while chloroform/methanol (1/1, *v/v*) polar lipids rich extract from *S. muticum* demonstrated radical scavenging activity [33]. Lipid extracts from brown algae containing glycolipids (GL) showed the capacity to suppress ROS in lipopolysaccharide (LPS)-stimulated RAW 264.7 macrophages [36], that were positively correlated with the oxidative stability of eicosapentaenoic acid (20:5 *n*-3) and stearidonic acid (18:4 *n*-3) PUFA in their GL forms, which encouraged further studies in seaweed polar lipids as source functional lipids.

The lipid content of brown seaweeds has usually been addressed by FA profiling. However, FA in seaweeds are mainly included in polar lipids related to GL, phospholipids (PL), and betaine lipids, that have been pinpointed by using low tech methods [37,38]. The characterization of detailed lipidomic signatures using omics approaches for brown seaweeds was only performed for *Fucus vesiculosus* [39] and *Saccharina latissima* [40] and has never been performed for these species.

The goal of this work was to characterize lipid extracts from of *B. bifurcata* and *S. muticum* in terms of polar lipid molecular components, the polar lipdome, and antioxidant activity, and to attempt to relate composition with bioactivity, by signaling components with previously reported biological activity. Therefore, we performed an in-depth analysis of the lipid composition of *B. bifurcata* and *S. muticum* through a liquid chromatography coupled to high resolution mass spectrometry approach. The survey of antioxidant activity of total lipids extracts from *B. bifurcata* and *S. muticum* was conducted through their free radical scavenging potential against 2,2-diphenyl-1-picrylhydrazyl (DPPH) and 2,2′-azino-bis-3-ethylbenzothiazoline-6-sulfonic acid (ABTS) radicals. Overall, this work will contribute to the valorization of these two species, highlighting the potential of turning *S. muticum* biomass in particular into natural high-value products and commercially viable resources.

## 2. Materials and Methods

### 2.1. Reagents

Dichloromethane (CH_2_Cl_2_), methanol (MeOH), and acetonitrile (high-performance liquid chromatography—HPLC, grade) were purchased from Fisher Scientific Ltd. (Loughborough, UK). Milli-Q water (Synergy, Millipore Corporation, Billerica, MA, USA) was used. Phospholipid standards: 1,2-dimyristoyl-sn-glycero-3- phosphocholine (dMPC), 1,2-dimyristoyl-sn-glycero-3-phosphoethanolamine (dMPE), 1,2-dimyristoyl-sn-glycero-3-phospho-(1′-rac-)glycerol (dMPG), 1,2-dipalmitoyl-sn-glycero-3-phosphatidylinositol (dPPI), 1–nonadecanoyl-2-hydroxy-sn-glycero-3-phosphocholine (LPC), dimyristoyl phosphatidic acid (dMPA), and N-heptadecanoyl-D-erythro-sphingosine (Cer (d18:1/17:0)) were purchased from Avanti Polar Lipids, Inc. (Alabaster, AL).

2,2-Diphenyl-1-picrylhydrazyl (DPPH) was purchased from Aldrich (Milwaukee, WI). 2,2’-Azino-bis(3-ethylbenzothiazoline-6-sulfonic acid) diammonium salt (ABTS) was obtained from Fluka (Buchs, Switzerland). Ammonium acetate and 6-hydroxyl-2,5,7,8-tetramethylchromane-2-carboxylic acid (Trolox) were purchased from Sigma-Aldrich (St Louis, MO, USA). All other reagents and chemicals used were of the highest grade of purity and were purchased from major commercial sources.

### 2.2. Sampling

Bifurcaria bifurcata and S. muticum samples were collected in Aguda beach, Porto, Portugal (Portugal, 41°2′38″ N, 8°39′10″ W) on 18 April 2018 (spring). Biomass samples (composite sampling of at least five specimens were used) were washed thoroughly with fresh water to remove epiphytes, eliminate salt, sand, or shells and freeze-dried. Samples were maintained at −80 °C for posterior analyses.

### 2.3. Lipid Extraction

The biomass samples were extracted using a modified Bligh and Dyer method [41]. Samples were weighed (250 mg, a total of five replicates for each seaweed) and transferred to glass tubes with Teflon-lined screw caps. Total lipid extraction was performed by adding 3.75 mL of a mixture methanol:dichloromethane (2:1) to each replicate sample. After individual homogenization during 2 min using a vortex and 1 min of sonication, samples were incubated on ice on a rocking platform shaker (Stuart Scientific STR6, Bibby, UK) for 2 h and 30 min. The mixture was centrifuged at 392× *g* for 10 min (Pro-Analytical series, UK), and the organic phase was collected to a new glass tube. The remaining biomass residue was re-extracted three times with 3 mL of a mixture methanol:dichloromethane (2:1). Water (2 mL) was then added to the total collected organic phase and tubes were centrifuged again at 392× *g* for 10 min, and the lower organic phase was recovered. The remaining organic solvent was dried under a nitrogen gas stream and the total lipid extract content was estimated by gravimetry. Lipid extracts were stored at −20 °C, under nitrogen atmosphere until their use in liquid chromatography-mass spectrometry (LC–MS), gas chromatography-mass spectrometry (GC–MS) and bioactivity analyses [39,42].

### 2.4. Phospholipid and Glycolipid Quantification

Phospholipids quantification was performed using modified Bartlett and Lewis method as previously described [43]. At least, five replicates of 100 μL of lipid extract in dichloromethane (1 mg mL^−1^) were used. Absorbance of standards and samples was measured on a microplate UV-Vis spectrophotometer (Multiskan GO, Thermo Scientific, Hudson, NH, USA). Phospholipids were calculated as P x 25. Glycolipids quantification was performed by calculating the hexose content (% glucose) by the orcinol colorimetric method as described previously (CyberLipids, [43]). The amount of sugar was estimated from a calibration curve prepared by performing the reaction with defined amounts of glucose (up to 40 μg, from an aqueous solution containing 2 mg mL^−1^ of sugar) At least five replicates of 50 μL of lipid extract in dichloromethane (1 mg mL^−1^) were used. The factor 100/35 was chosen to convert glucose to GL [43,44].

### 2.5. Fatty Acid Analysis by Gas Chromatography-Mass Spectrometry (GC–MS)

Fatty acid methyl esters (FAME) were prepared from total lipid extracts by alkaline transesterification using a methanolic solution of potassium hydroxide (2.0 M), according to the Aued-Pimentel-based methodology, as described for seaweeds [43]. Therefore, 1 mL of internal standard (1 μg mL^−1^ of methyl nonadecanoate (C19:0) in n-hexane) was added to 30 µg of total lipid extract in dichloromethane (1 mg mL^−1^), followed by 200 µL of potassium hydroxide (2.0 M), prepared in methanol. After 2 min of vortexing, 2 mL of NaCl aqueous solution (10 g L^−1^) was added. The mixture was centrifuged at 392× *g* for 5 min, and 600 µL of organic phase were collected and dried under a nitrogen gas stream. For GC–MS analysis, the derivatized extract was diluted in 60 µL of hexane. Sample volumes of 2.0 µL of the hexane solution containing FAME were analyzed by GC-MS on an Agilent Technologies 6890 N Network (Santa Clara, CA, USA) equipped with a DB-FFAP column with the following specifications: 30 m of length, 0.32 mm internal diameter, and 0.25 µm film thickness (123-3232, J&W Scientific, Folsom, CA, USA). The GC equipment was connected to an Agilent 5973 Network Mass Selective Detector operating with an electron impact mode at 70 eV and scanning the range *m/z* 50–550 in a 1 s cycle in a full scan mode acquisition. The oven temperature was programed at an initial temperature of 80 °C for 3 min followed by a linear increase to 160 °C and 10 min at this temperature. Helium was used as carrier gas at flow rate of 1.4 mL min^−1^. Two analytical replicates of four lipid extracts (total of eight replicates, *n* = 2 × 4) were injected into the equipment. The identification of each FA was performed considering the retention times (Figure S1) and similarity to MS spectra of FA standards (Supelco 37 Component Fame Mix, Sigma-Aldrich, St. Louis, MO, USA) and available spectra in the Wiley 275 library and AOCS Lipid Library. The relative amounts of FA were calculated by the percent relative area method with proper normalization using C19:0 as internal standard, considering the sum of all relative areas of the identified FA. Results were expressed as means ± standard deviation (SD).

### 2.6. Polar Lipid Analysis by Hydrophilic Interaction Liquid Chromatography-High Resolution Mass Spectrometry (HILIC−MS) and Tandem Mass Spectrometry (MS/MS)

Lipid extracts analysis was performed in a HPLC Ultimate 3000 Dionex (Thermo Fisher Scientific, Bremen, Germany) system with an autosampler and coupled online to the Q-Exactive^®^ hybrid quadrupole Orbitrap^®^ mass spectrometer (Thermo Fisher Scientific, Waltham, MA, USA). The solvent system consisted of two mobile phases as follows: mobile phase A (acetonitrile:methanol 60:40 (per volume) with 2.5 mM ammonium acetate) and mobile phase B (acetonitrile:methanol:water 50:25:25 (per volume) with 2.5 mM ammonium acetate). Initially, 90% of mobile phase A was held isocratically for 2 min, followed by a linear decrease to 10% of A within 13 min, after which settings were maintained for 2 min and then returned to the initial conditions in 3 min, followed by a re-equilibration period of 10 min prior to the next analysis. A volume of 5 µL of each sample, containing 5 µg of lipid extract, a volume of 4 µL of PL standards mix (dMPC—0.02 µg, dMPE—0.02 µg, lysophosphatidylcholine (LPC)—0.02 µg, dPPI—0.08 µg, dMPG—0.012 µg, dMPA—0.08 µg, Cer—0.04 µg) and 91 µL of eluent (10% of mobile phase A and 90% of mobile phase B) were mixed and introduced into the Ascentis^®^Si column (10 cm × 1 mm, 3 µm, Sigma-Aldrich) with a flow rate of 50 µL min^−1^ and at 35 °C.

The mass spectrometer was operated simultaneously in positive (electrospray voltage 3.0 kV) and negative (electrospray voltage −2.7 kV) modes with a resolution of 70,000 and automatic gain control (AGC) target of 1 × 10^6^, the capillary temperature was 250 °C and the sheath gas flow was 15 U. In MS/MS experiments, a resolution of 17,500 and AGC target of 1 × 10^5^ were used. Cycles consisted of one full scan mass spectrum and 10 data-dependent MS/MS scans were repeated continuously throughout the experiments with the dynamic exclusion of 60 s and intensity threshold of 2 × 10^4^ [39,42]. Normalized collision energy (CE) ranged between 20, 25, and 30 eV. Four replicates (corresponding to four lipid extracts, *n* = 4) were analyzed.

### 2.7. Data Analysis

Data acquisition was carried out using the Xcalibur data system (V3.3, Thermo Fisher Scientific, Waltham, MA, USA). Peak integration and assignments of HPLC–MS data were performed using MZmine 2.39. The software was used for filtering and smoothing, peak detection, peak processing, and assignment against an in-house database. The validated peaks were within the time range of a MS full run. All the peaks with intensity lower than 1 × 10^4^ were excluded. All the information originating from the MZmine software was confirmed based on the assignment of the molecular ions observed in the LC–MS spectra, typical retention time, exact mass accuracy, and MS/MS spectra information. Only exact mass accuracy with an error of less than 5 ppm was considered. MS/MS spectra were performed to confirm the identity of the molecular species as previously described by Marine Lipidomics Laboratory group [39,45,46]. The identification of molecular species of polar lipids was based on the LC retention time (Tables S1 and S2, Figure S2). The normalization of the identified lipid species was performed by exporting integrated peak areas values (.csv file) and dividing the peak area value of each species by the peak area value of a standard lipid species with the closest retention time. The resulting data matrix with normalized areas of all species was used to relative quantitation of each lipid species per class, by dividing each normalized peak area by the sum of all normalized peak areas of the lipid species (ions) assigned in each class and multiplying by 100 to get the relative abundance in percentage. Data was exported into software GraphPad Prism 8.0 to design bar graphs. Identified lipid species were subjected to further analysis for determination of common and unique species through Venn diagram representation using the open source jvenn [47]. We defined “unique” when a lipid species was found only in *B. bifurcata* or *S. muticum* datasets. “Common” lipid species are those that have been found in samples from both seaweeds (Table S3).

2.8. 2.2´-Azino-bis-3-Ethylbenzothiazoline-6-Sulfonic Acid Radical Cation (ABTS) Assay Radical Scavenging Activity

The antioxidant scavenging activity against 2,2´-azino-bis-3-ethylbenzothiazoline-6-sulfonic acid radical cation (ABTS^●+^) was evaluated using a previously described method [42] with some modifications. The ABTS radical solution (3.5 mmol L^−1^) was prepared by mixing 10 mL of ABTS stock solution (7 mmol L^−1^ in water) with 10 mL of potassium persulfate K_2_S_2_O_8_ (2.45 mmol L^−1^ in water). This mixture was kept for 12–16 h in the dark at room temperature and was diluted in ethanol to obtain an absorbance value of ≈0.9 measured at 734 nm using a UV-vis spectrophotometer (Multiskan GO 1.00.38, Thermo Scientific, Hudson, NH, USA). For an evaluation of the radical stability, a volume of 150 µL of ethanol was added to 12 microplate wells followed by addition of 150 µL of ABTS^●+^ diluted solution and an incubation period of 120 min, with absorbance measured at 734 nm every 5 min. For an evaluation of the radical scavenging potential, a volume of 150 µL of *S. muticum* (5–250 µmol L^−1^ in ethanol) and *B. bifurcata* (25–250 µmol L^−1^ in ethanol) lipid extracts and 150 µL of Trolox standard solution (10–75 µmol L^−1^) were placed in each well followed by addition of 150 µL of ABTS^●+^ diluted solution, and absorbance was measured at 734 nm every 5 min during a total incubation period of 120 min. Control lipid assays were performed by replacing 150 µL of ABTS^●+^ diluted solution by 150 µL of ethanol. Radical reduction was monitored by measuring the decrease in absorbance during the reaction, thereby quantifying radical scavenging activity. All measurements were performed in triplicate. The % of ABTS radical remaining was determined according to Equation (1):

% ABTS remaining = (Abs samples after incubation time/Abs sample at the beginning of reaction) × 100 (1)

The free radical-scavenging activity of samples was determined as the percentage of inhibition of ABTS radical according to Equation (2):

% Inhibition = ((Abs ABTS − (Abs samples − Abs control))/Abs ABTS) × 100 (2)

The concentration of samples reducing 50% of ABTS radical after 120 min (IC_50_) were calculated by linear regression using the concentration of samples and the percentage of the inhibition curve. The activity, expressed as Trolox Equivalents (TE, µmol Trolox/g of sample), was calculated according to Equation (3):

TE = IC_50_ Trolox (µmol L^−1^) × 1000/IC_50_ of samples (µg mL^−1^) (3)

### 2.9. 2.2-Diphenyl-1-Picrylhydrazyl Radical Assay (DPPH) Radical Scavenging Activity

The antioxidant scavenging activity against ⍺,⍺-diphenyl-𝛽-picrylhydrazyl radical (DPPH^●^) was evaluated using a previously described method applied with some modifications [42,47]. A stock solution of DPPH^●^ in ethanol (250 µmol L^−1^) was prepared and diluted to provide a working solution with an absorbance value of ≈0.9 measured at 517 nm using a UV-Vis spectrophotometer (Multiskan GO 1.00.38, Thermo Scientific, Hudson, NH, USA). To evaluate the radical stability, a volume of 150 µL of ethanol was added to 12 microplate wells followed by addiction of 150 µL of DPPH^●^ diluted solution and an incubation period of 120 min, with absorbance measured at 517 nm every 5 min. For evaluation of the radical scavenging potential, a volume of 150 µL of *S. muticum* and *B. bifurcata* lipid extracts (25–250 µmol L^−1^) and 150 µL of Trolox standard solution (10–75 µmol L^−1^) were placed in each well followed by addition of 150 µL of DPPH^●^ diluted solution, and again an incubation period of 120 min was followed with absorbance being measured at 517 nm every 5 min. Control lipid assays were performed by replacing 150 µL of DPPH^●^ diluted solution by 150 µL of ethanol. Radical reduction was monitored by measuring the decrease in absorbance during the reaction, thereby quantifying radical scavenging activity. All measurements were performed in triplicate. The % of DPPH radical remaining was determined according to Equation (4):

% DPPH remaining = (Abs samples after incubation time/Abs sample at the beginning of reaction) × 100 (4)

The free radical-scavenging activity of samples was determined as the percentage of inhibition of DPPH radical according to Equation (5):

% Inhibition = ((Abs DPPH − (Abs samples − Abs control))/Abs DPPH) × 100 (5)

The concentration of samples reducing 50% of DPPH radical after 120 min (IC_50_) was calculated by linear regression using the concentration of samples and the percentage of the inhibition curve. For *B. bifurcata* the IC_25_ was calculated instead of IC_50_. The activity expressed, as TE (µmol Trolox/g of sample), was calculated according to Equation (3) (for *S. muticum*) and Equation (6) (for *B. bifurcata*):

TE = IC_25_ Trolox (µmol L^−1^) × 1000/IC_25_ of samples (µg mL^−1^) (6)

## 3. Results

### 3.1. Total Lipid Content

The total lipid content of the two brown seaweeds, *B. bifurcata* and *S. muticum,* was estimated by gravimetry after lipid extraction procedure. The average lipid content (expressed as g/100 g of dry weigh biomass, DW) of *B. bifurcata* was 7.20 ± 0.31 g/100 g while that of *S. muticum* was lower, representing 2.80 ± 0.06 g/100 g. *Bifurcaria bifurcata* presented a total of 0.12 ± 0.03 g/100 g^1^ of PL and 1.01 ± 0.07 g/100 g of GL. *Sargassum muticum* presented higher content of both PL and GL in lipid extracts, with 0.25 ± 0.03 g/100 g and 1.31 ± 0.04 g/100 g, respectively.

#### 3.1.1. *Bifurcaria bifurcata* and *S. muticum* Fatty Acid Profiles

The FA profile of the extracts obtained from *B. bifurcata* and *S. muticum* were determined by GC–MS analysis of the FAME obtained after alkaline transmethylation [45] (Table 1). In the case of *B. bifurcata*, the fatty acid profile included 16 FA species, and the most abundant was 16:0 (30.07% ± 2.78%), followed by 20:4 *n*-6 (14.28% ± 0.72%) and 18:1 *n*-9 (12.12% ± 1.22%). The lipid fraction of this seaweed is rich in saturated fatty acids (SFA) (46.09% ± 4.30%), with 16:0 and 18:0 being the major contributors for total SFA. The monounsaturated fatty acids (MUFA) included 16:1, 18:1 *n*-9, and 20:1, and the PUFA included FAs 18:2 *n*-6, 18:3 *n*-3, 18:4 *n*-3, 20:3, 20:4 *n*-6, and 20:5 *n*-3 (Table 1). The results obtained for the *S. muticum* allowed identifying 20 different FA (Table 1). The most abundant was 16:0 with a relative content of 24.18% ± 0.48%, followed by 20:4 *n*-6 (12.53% ± 0.72%) and 20:5 *n*-3 (9.71% ± 0.34%). This brown seaweed exhibited a FA profile rich in PUFA, with 16:2, 18:2 *n*-6, 18:3 *n*-3, 18:4 *n*-3, 20:2, 20:3, 20:4 *n*-6, and 20:5 *n*-3 as the major contributors for total PUFA abundance. MUFA such as 16:1, 18:1 *n*-9, 20:1, and 22:1, and SFA such as 14:0, 15:0, 16:0, 18:0, 20:0, 22:0 were also identified (Table 1). PUFA content of *B. bifurcata* was lower than that of *S. muticum.*

#### 3.1.2. *Bifurcaria bifurcata* and *S. muticum* Polar Lipid Profiles

The polar lipid profiles of *B. bifurcata* and *S. muticum* were characterized at the molecular level by high resolution HILIC-LC–MS and MS/MS analysis. In the case of *B. bifurcata*, this approach allowed identifying 143 lipid species, from nine classes of polar lipids. Polar lipid profile included 68 GL species distributed into three GL classes, 44 PL species distributed into four PL classes and 31 betaine lipids species distributed into two classes (Table 2). Considering GL profile, in *B. bifurcata*, the GL classes identified included the acidic glycolipid sulfoquinovosyl diacylglycerol class (SQDG), as well as the neutral glycolipids digalactosyl diacylglycerol (DGDG) and monogalactosyl diacylglycerol (MGDG) classes (Table 2 and Table S1 from Supplementary material) but no lyso-forms were identified.

In the *S. muticum* lipid extracts a total of 217 polar lipid species were identified, distributed into 13 lipid classes. A total of 94 species of GL distributed into four different classes were identified, as well as 73 PL species distributed into seven classes, and 50 betaine lipid species distributed in two classes (Table 2). The criteria for identifying the lipid species included accuracy of the mass measurements (< 5 ppm), the LC retention time and the characteristics of the MS/MS spectra for each lipid class.

In what concerned GL profile, the classes of SQDG, of DGDG, and MGDG were identified in both algae, while lyso forms monogalactosyl monoacylglycerol (MGMG) were only seen in *S. muticum* (Table 2 and Table S2 from Supplementary material).

**Figure 1:**
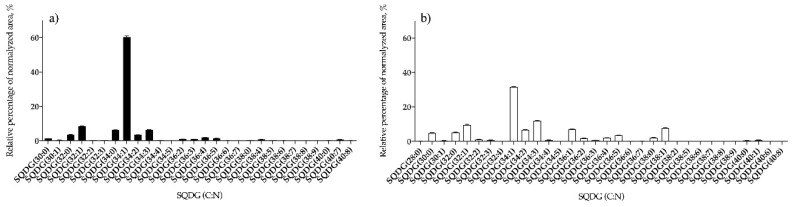
Relative abundance (RA) of lipid species of the sulfoquinovosyl diacylglycerol (SQDG) class identified in *B. bifurcata* (**a**) and *S. muticum* (**b**). The results are expressed as relative percentages after dividing the normalized peak area of each lipid species to the sum of normalized peak areas for all the lipid species within the class obtained after LC–MS analysis. Numbers in parentheses (C:N) indicate the number of carbon atoms (C) and double bounds (N) in the fatty acid side chains. The species with higher RA was SQDG (34:1) for both seaweeds.

These acidic SQDGs were identified as [M − H]^−^ and [M + NH_4_]^+^ in LC-MS spectra and typical fragmentation observed in LC–MS/MS spectra is shown in Figure S3a,b in the Supplementary material, respectively. Overall, 28 and 32 lipid species of SQDG were identified for *B. bifurcata* and *S. muticum*, respectively (Figure 1, Tables S1 and S2). The most abundant SQDG was assigned as SQDG (34:1) at *m/z* 819.5, for both seaweed species, being identified as SQDG (14:0/20:1), SQDG (16:0/18:1), and SQDG (16:1/18:0) in *B. bifurcata* (Figure 1a, Table S1), and as SQDG (16:0/18:1) in *S. muticum* (Figure 1b, Table S2).

**Figure 2:**
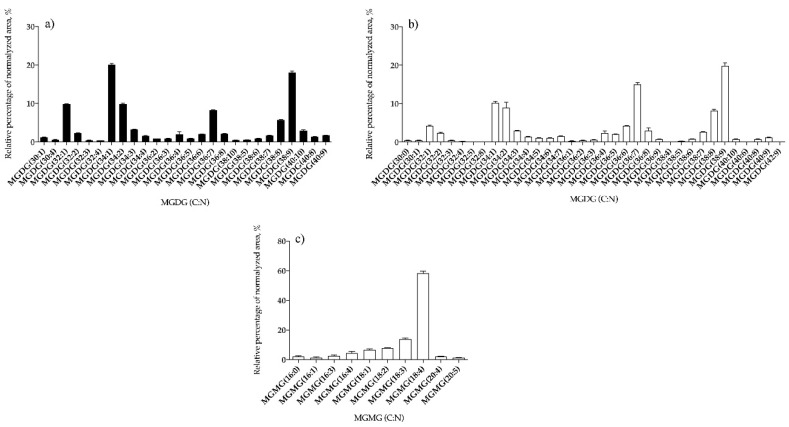
Relative abundances (RA) of lipid species of monogalactosyl diacylglycerol (MGDG) identified in *B. bifurcata* (**a**) and *S. muticum* (**b**) and of monogalactosyl monoacylglycerol (MGMG) identified in *S. muticum* (**c**). The results are expressed as relative percentage after dividing the normalized peak area of each lipid species to the sum of normalized peak areas for all the lipid species within the class obtained after LC–MS analysis. Numbers in parentheses (C:N) indicate the number of carbon atoms (C) and double bounds (N) in the fatty acid side chains. The species with higher RA were MGDG (34:1) for *B. bifurcata* and MGDG (38:9) and MGMG (18:4) for *S. muticum*.

**Figure 3:**
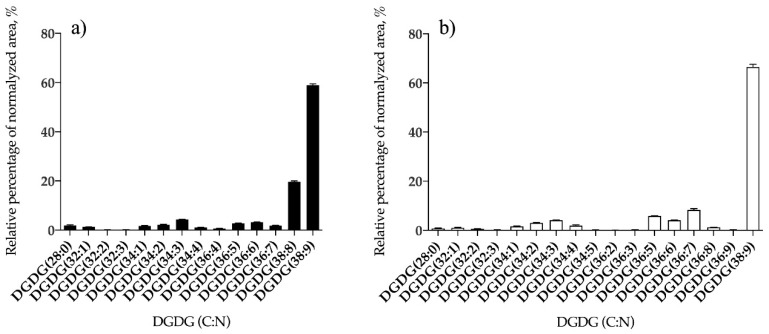
Relative abundances (RA) of lipid species of digalactosyl diacylglycerol (DGDG) glycolipids identified in *B. bifurcata* (**a**) and *S. muticum* (**b**). The results are expressed as relative percentage after dividing the normalized peak area of each lipid species to the sum of normalized peak areas for all the lipid species within the class obtained after LC–MS analysis. Numbers in parentheses (C:N) indicate the number of carbon atoms (C) and double bounds (N) in the fatty acid side chains. The species with higher RA was DGDG (38:9) for both seaweeds.

The neutral GL were detected in the positive LC–MS spectra as [M + NH4]+ ions (Tables S1 and S2) and the typical fragmentation obtained in the LC–MS/MS spectra of MGDG, MGMG, and DGDG species as [M + NH_4_]^+^ are shown in Figure S4 for MGDG and MGMG and Figure S5 for DGDG species. Considering the neutral GL, 26 species of MGDG (Figure 2a and Table S1) and 14 of DGDG (Figure 3a and Table S1) were identified in *B. bifurcata* and 35 lipid species of MGDG (Figure 2b and Table S2), 10 of MGMG (Figure 2c and Table S2), and 17 of DGDG (Figure 3b and Table S2) were identified in *S. muticum*. In *B. bifurcata* the most abundant MGDG was MGDG (34:1), at *m/z* 774.6, and assigned as MGDG (16:0/18:1), while in *S. muticum* the most abundant was MGDG (38:9) at *m/z* 814.5, assigned as MGDG (20:5/18:4) (Figure 2, Tables S1 and S2). The DGDG (38:9) at *m/z* 976.6 was the most abundant DGDG in both seaweed species and was identified as DGDG (20:5/18:4) also for both (Figure 3, Tables S1 and S2). The most abundant MGMG in *S. muticum* was detected at *m/z* 530.3 and was assigned as MGMG (18:4) (Figure 2c and Table S2). The profile for each GL class is represented in Figure 2 and Figure 3, showing the relative abundance of the lipid species per class.

**Figure 4:**
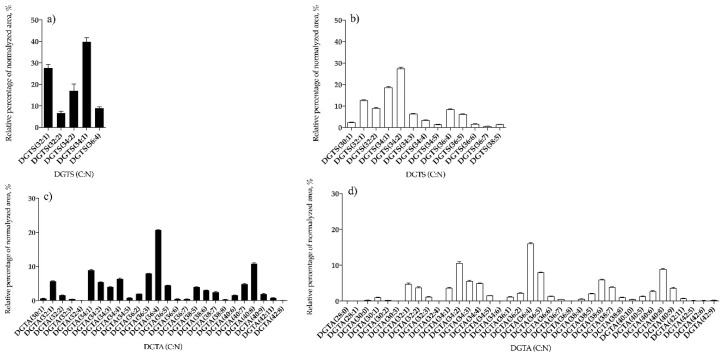
Relative abundance (RA) of betaine lipid species identified as diacylglyceroltrimethylhomoserine (DGTS) (**a**) and diacylglyceroltrimethyl-β-alanine (DGTA) (**c**) in *B. bifurcata*, and DGTS (**b**) and DGTA (**d**) in *S. muticum*. The results are expressed as relative percentage after dividing the normalized peak area of each lipid species to the sum of normalized peak areas for all the lipid species within the class obtained after LC–MS analysis. Numbers in parentheses (C:N) indicate the number of carbon atoms (C) and double bounds (N) in the fatty acid side chains. The species with higher RA were DGTS (34:1) and DGTA (36:4) for *B. bifurcata* and DGTS (34:2) and DGTA (36:4) for S. *muticum*.

Betaine lipids identified in *B. bifurcata* and *S. muticum* were assigned to the diacylglyceroltrimethylhomoserine (DGTS) and diacylglyceroltrimethyl-β-alanine (DGTA) classes, which were identified in the LC–MS spectra in positive ion mode, as [M + H]^+^ ions. The DGTA class is a structural isomer of DGTS and both were discriminated on the basis of having different retention times [39]: DGTA molecular species eluted at RT 9 min while DGTS molecular species are eluted at RT 6 min. Overall, five lipid species of DGTS (Figure 4a, Table S1) and 26 lipid species of DGTA (Figure 4c and Table S1) were found in *B. bifurcata*, and 13 lipid species of DGTS (Figure 4b, Table S2) and 37 lipid species of DGTA (Figure 4d, Table S1) were present in *S. muticum*. The predominant DGTS species in *B. bifurcata* was detected at *m/z* 738.6, corresponding to DGTS (34:1) (Figure 4a and Table S1) while in *S. muticum* the predominant DGTS was detected at *m/z* 736.6, corresponding to DGTS (34:2), (Figure 4b, Table S2). DGTA assigned as DGTA (36:4) at *m/z* 760.6 had similar predominance for both seaweed species (Figure 4c,d, and Tables S1 and S2). The structural features of betaine lipids were confirmed through the identification of the typical product ions and fragment pathways observed in the LC–MS/MS spectra (Figure S6).

**Figure 5:**
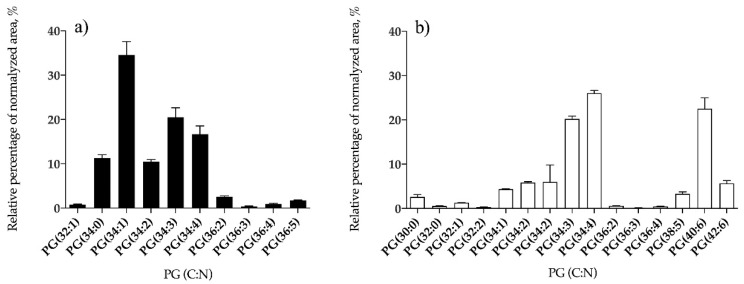
Relative abundance (RA) of lipid species of the phosphatidylglycerol (PG) class identified in *B. bifurcata* (**a**) and *S. muticum* (**b**). The results are expressed as relative percentage after dividing the normalized peak area of each lipid species to the sum of normalized peak areas for all the lipid species within the class obtained after LC–MS analysis. Numbers in parentheses (C:N) indicate the number of carbon atoms (C) and double bounds (N) in the fatty acid side chains. The species with higher RA were PG (34:1) for *B. bifurcata* and PG (34:4) for *S. muticum*.

**Figure 6:**
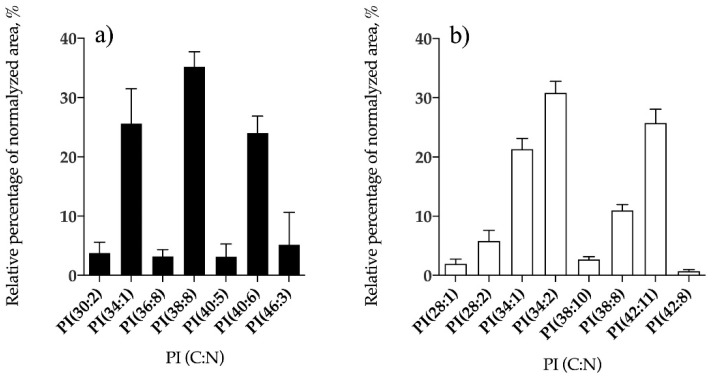
Relative abundance (RA) of lipid species of the phosphatidylinositol (PI) class identified in *B. bifurcata* (**a**) and *S. muticum* (**b**). The results are expressed as relative percentage after dividing the normalized peak area of each lipid species to the sum of normalized peak areas for all the lipid species within the class obtained after LC–MS analysis. Numbers in parentheses (C:N) indicate the number of carbon atoms (C) and double bounds (N) in the fatty acid side chains. The species with higher RA were PG (38:8) for *B. bifurcata* and PI (34:2) for *S. muticum*.

**Figure 7:**
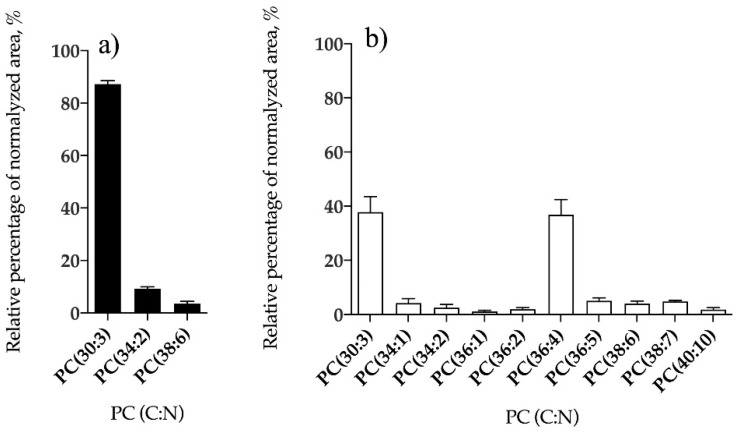
Relative abundance (RA) of lipid species of the phosphatidylcholine (PC) class identified in *B. bifurcata* (**a**) and *S. muticum* (**b**). The results are expressed as relative percentage after dividing the normalized peak area of each lipid species to the sum of normalized peak areas for all the lipid species within the class obtained after LC–MS analysis. Numbers in parentheses (C:N) indicate the number of carbon atoms (C) and double bounds (N) in the fatty acid side chains. The species with higher RA were PC (30:3) for *B. bifurcata,* and PC (30:3) and PC (36:4) for *S. muticum*.

**Figure 8:**
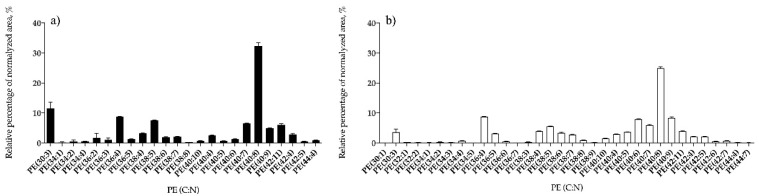
Relative abundance (RA) of lipid species of the phosphatidylethanolamine (PE) class identified in *B. bifurcata* (**a**) and *S. muticum* (**b**). The results are expressed as relative percentage after dividing the normalized peak area of each lipid species to the sum of normalized peak areas for all the lipid species within the class obtained after LC–MS analysis. Numbers in parentheses (C:N) indicate the number of carbon atoms (C) and double bounds (N) in the fatty acid side chains. The species with higher RA was PC (40:8) for both seaweeds.

Phospholipid classes identified in *B. bifurcata* and *S. muticum* included phosphatidylcholine (PC), phosphatidylethanolamine (PE), phosphatidylglycerol (PG), and phosphatidylinositol (PI). *Sargassum muticum* also contained lysoPC, lysoPE, and lysoPG species, while no lyso-forms of PL were identified in *B. bifurcata* (Table 2). Overall, 10 lipid species of PG (Figure 5a, Table S1), seven PI (Figure 6a, Table S1), three PC (Figure 7a, Table S1), and 24 PE (Figure 8a, Table S1) were identified in *B. bifurcata*. On the other hand, in *S. muticum,* 15 species of PG (Figure 5b, Table S2), one LPG (Table S2), eight PI (Figure 6b, Table S2), 10 PC (Figure 7b, Table S2), three LPC (Table S2), 34 of PE (Figure 8b, Table S2), and two of lysophosphatidylethanolamine (LPE) (Table S2) were identified. The predominant PG of *B. bifurcata* was assigned as PG (34:1) at *m/z* 747.5 (Figure 5a and Table S1) while the predominant PG of *S. muticum* was assigned as PG (34:4) at *m/z* 741.5 (Figure 5b and Table S2). PG and lysophosphatidylglycerol (LPG) classes were identified in negative LC−MS spectra as [M − H]^−^ ions and respective typical MS/MS spectra are represented in Figure S7a,b. The predominant PI of *B. bifurcata* was assigned as PI (38:8) at *m/z* 877.5 (Figure 6a and Table S1) while the predominant PI of *S. muticum* was assigned as PI (34:2) at *m/z* 833.5 (Figure 6b and Table S2). The PI class was identified in negative LC–MS spectra as [M − H]^−^ ions, and a characteristic MS/MS spectrum is shown in Figure S5c. LPC, PC, LPE, and PE molecular species were identified in positive LC–MS spectra as [M + H]^+^ ions. The predominant PC for *B. bifurcata* was assigned as PC (30:3) at *m/z* 700.5 (Figure 7a, Table S1), while the predominant PC species for *S. muticum* were PC (30:3) at *m/z* 700.5 and PC (36:4) at *m/z* 782.6 (Figure 7b and Table S2). The predominant PE for both seaweed species was assigned as PE (40:8) at *m/z* 788.5 (Figure 8a,b, Tables S1 and S2). Figure S8 shows the typical fragmentation pathways observed in the LC-MS/MS spectra of PC, LPC, PE, and LPE.

We compared the common and contrasting lipid species in the lipidome of these two brown seaweeds, members of the *Fucales* order. We found 124 lipid species that were common (52.5% of total number of lipid species), 19 lipid species found only in the lipidome of *B. bifurcata* (8.1%), and 93 lipid species identified only in the lipidome of *S. muticum* (39.4%). The common lipid species included 24 MGDG, 25 SQDG, 12 DGDG species, three PC, 22 PE, eight PG, and two PI species and five DGTS and 23 DGTA species (Table S3). In the case of *B. bifurcata*, there were two MGDG, two DGDG, three SQDG, two PE, two PG, five PI, and three DGTA unique species. Many lipid species were only assigned to *S. muticum* lipidome and included 11 MGMG, five DGDG, seven SQDG, seven PC, 12 PE, seven PG, six PI, and betaine lipids (eight DGTS, and 14 DGTA). Moreover, 10 MGMG, three LPC, two LPE, and one LPG were only found in *S. muticum* lipidome and not in *B. bifurcata,* since these classes were not detected in the latter.

### 3.2. Antioxidant Activity

**Figure 9:**
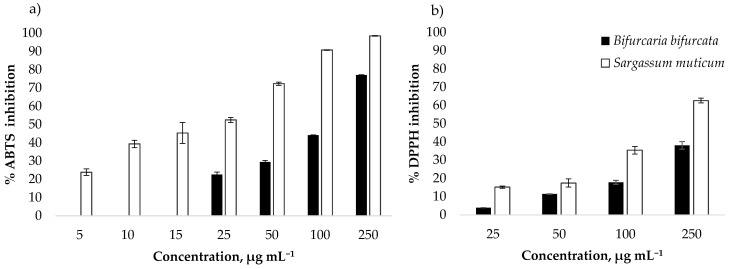
Free radical-scavenging activity (%) on 2,2´-azino-bis-3-ethylbenzothiazoline-6-sulfonic acid radical cation (ABTS) (**a**) and 2,2-diphenyl-1-picrylhydrazyl radical (DPPH) assays (**b**) radicals of *B. bifurcata* (**a**) and *S. muticum* lipid extracts. Each value is expressed as mean ± standard deviation (*n* = 3).

The antioxidant activity of *B. bifurcata* and *S. muticum* lipid extracts was evaluated by 2,2´-azino-bis-3-ethylbenzothiazoline-6-sulfonic acid radical cation assay (ABTS^●+^) and 2,2-diphenyl-1-picrylhydrazyl radical assay (DPPH^●^) that measured the free radical scavenging capacity of the lipid extracts. The percentage of radical inhibition in the presence of lipid extracts was calculated after 120 *min*. Seven varying concentrations of lipid extracts ranging from 5 to 250 μg mL^−1^ of *S. muticum* and four varying concentrations ranging from 25 to 250 μg mL^−1^ of *B. bifurcata* were tested for ABTS antioxidant activity, while concentrations ranging from 25 to 250 μg mL^−1^ of *B. bifurcata* and *S. muticum* were tested for DPPH antioxidant activity. A dose-dependent increase in the scavenging capacity was observed for all concentrations tested (Figure 9). The 250 μg mL^−1^ extracts showed the best antioxidant activity, where among them, the *S. muticum* extract showed the highest antioxidant potential both in ABTS (98.4% ± 0.19%) and DPPH (62.59% ±1.29%).

For *B. bifurcata*, 50% of inhibition (IC_50_) of ABTS^●+^ radical was attained at a concentration of 134.19 ± 2.12 μg mL^−1^(IC_50_), representing a TE of 100.33 ± 1.57 µmol g^−1^, while for DPPH^●^, only 25% of inhibition (IC_25_) was attained at a concentration of 155.00 ± 3.96 μg mL^−1^ representing a TE of 74.89 ± 1.93 µmol g^−1^ (Table 3). *Sargassum muticum* lipid extracts showed antioxidant activity against both DPPH^●^ and ABTS^●+^ radicals (Table 3). For ABTS^●+^ radical scavenging activity, IC_50_ was attained at 23.42 ± 0.79 μg mL^−1^, representing a TE of 648.35 ± 21.43 µmol g^−1^ and for DPPH assay the IC_50_ was attained at a concentration of 188.43 ± 2.55 μg mL^−1^ representing a TE of 124.19 ± 14.39 µmol g^−1^ (Table 3).

## 4. Discussion

Seaweeds are currently recognized as promising sources of different bioactive metabolites, making them attractive for prospection efforts in the search of new functional ingredients and pharmacophores/drugs [21]. In this work we studied the lipid composition and antioxidant activity of *S. muticum,* an invasive species of eastern European shores. The invasive *S. muticum* species may represent an especially attractive prospective resource because of its abundance and wide availability, and also because of the prospect of removing its biomass from the invaded habitats. In order to study the promise and pertinence of using *S. muticum* as an industry resource, we compared its composition and antioxidant potential to those of a native species of the Iberian Atlantic coast inhabiting the same geographic niche for some decades, in *B. bifurcata*. Commercially viable solutions such as the use of lipids from brown seaweeds as source of functional lipids for food and cosmetic, pharmaceutical products could encourage their harvesting, which became particularly relevant in the case of invasive species. To our knowledge, the present study represents the first detailed characterization of the lipidomic signature of these two brown seaweeds, *B. bifurcata* and *S. muticum,* from the Portuguese coast. The lipid content obtained for *B. bifurcata* was 7.29% ± 0.51% DW, while for *S. muticum* it was 2.84% ± 0.16% DW. These values were similar with the ones reported in the literature for both seaweeds, *B. bifurcata* [7,25] and *S. muticum* [27,33,48].

Both seaweeds showed the presence of SFA in their FA profile, namely 16:0, MUFA and PUFA, namely 18:1 *n*-9, 18:4 *n*-3, 20:4 *n*-6, and 20:5 *n*-3, respectively, in accordance with the available literature for the FA composition of *B. bifurcata* [7,24,25] and *S. muticum* [26,27]. Both seaweeds attract specific interest as edible seaweeds. The low *n*-6/*n*-3 ratio found in both algae guarantees appealing nutritional and health characteristics, since *omega*-3 FA are proposed to be beneficial for the prevention of cardiovascular diseases and other chronic diseases [21,49], while also being associated with antimicrobial, antiviral, anti-inflammatory, and antitumoral properties [29,50,51]. *Sargassum muticum* displayed a higher PUFA content and *n*-3 FA, unveiling a profitable avenue for further research in the nutraceutical/dietary supplements scope.

Polar lipids identified in the two seaweeds include GL, phospholipids, and betaine lipids. *Sargassum muticum* presented a much more varied and complex polar lipidome (217 vs. 143 different lipid species detected) making it a much more attractive target if searching for bioactive lipids. That happens in the case of GL, with *S. muticum* presenting 62 different lipid species from the class, while *B. bifurcata* only presented 40. GL from algae have especially been assigned bioactivities such as antioxidant, anti-inflammatory, antiviral, antibacterial, and antitumoral activities [46,52]. Besides glycolipids, phospholipids also represented a major component of the lipidome of *B. bifurcata* and *S. muticum*. Phospholipids are major components of cell membranes and can be important signaling molecules in living organisms, as well as in seaweeds [53]. In addition, considering seaweeds as foods or as raw materials for other biotechnological applications, the PL rich in PUFA can be potentially used as ingredients for supplements or for food fortification in *n*-3 FA, since there is evidence that PLs are better at delivering PUFA than the triglycerides from diet [42,46].

Betaines are also important components of brown seaweeds, although far less studied [39,40]. It was suggested that DGTS species have the same biological function as PC due to their similar zwitterionic structure, with both lipid classes being able to interchange their roles within the cell [54]. Some species of Phaeophyta can have DGTS and/or DGTA, the latter formed by conversion of DGTS into DGTA [55]. The Fucales order, to which *B. bifurcata* and *S. muticum* belong, are known to accumulate high amounts of DGTA/DGTS, while displaying reportedly low amounts of PC [39,56]. However, the bioactivities and nutritional value of the betaine lipids are yet to be unraveled.

The biological potential of polar lipids present in both lipidomes enhance the commercial viability to turn these seaweeds into components with value for different biotechnological applications and industries, as well as explored for compounds with bioactivities, representing viable options in the prevention and management of modern lifestyle diseases [57]. In this work, we explored the antioxidant activity of lipid extracts from both seaweeds, fostering their valorization. Total lipid extracts from *B. bifurcata* and *S. muticum* showed antioxidant activity potential in the µg mL^−1^ range. The values of IC_50_ for ABTS free radical scavenging assays obtained for the *B. bifurcata* lipid extract (Table 3), are comparable to ones reported by Santos et al. in dichloromethane extract [24] (ABTS^●+^, IC_50_ 116.25 ± 2.54 µg mL^−1^). Otherwise, slightly higher DPPH radical scavenging activity has been reported in methanol extract [7] and dichloromethane extracts [58], revealing the impact of extraction solvents selection in antioxidant activities, with different solvent polarities altering their efficacy to extract specific antioxidant components [31]. Therefore, our study contributes for the valorization *B. bifurcata* as a promising source of bioactive compounds and functional ingredients. *B. bifurcata* may be produced in integrated multi-trophic aquaculture setups, allowing taking advantage of lipid compounds of interest and, otherwise, promoting the aquaculture sector [24]. *Sargassum muticum* lipid extracts showed higher antioxidant activity than the ones from *B. bifurcata* in both ABTS and DPPH radical scavenging assays. The IC_50_ values presented by *S. muticum* for ABTS^●+^ assay (Table 3) are also lower than those presented in the literature for *S. serratifolium* ethyl acetate (IC_50_, 34.6 ± 0.47 μg mL^−1^), or methanol and ethanol extracts, mentioned as effective antioxidants [59]. These results concur to the realization of the great antioxidant potential of *S*. *muticum* extracts. The results of the DPPH assay in this survey also unveil a higher antioxidant capacity than that demonstrated for *S. muticum* chloroform/methanol extracts (1/1, *v/v*) meaning the extraction procedure used in our study is efficient in the capture of lipid compounds with antioxidant properties [33]. The reported findings also demonstrated that polar lipids can be relevant active lipid players in the antioxidant activity survey of the total lipid extract [33]. Overall, the results from antioxidant assays are more promising for *S. muticum* than for the native *B. bifurcata*, supporting the pertinence of using the biomass of this invasive species for production of bioactive liquid extracts, for further bioactivity prospection. Interestingly, from a structural point of view, a distinct lipid profile between both seaweeds highlighted by lipid species rich in PUFA and also the higher PL and GL content obtained in *S. muticum* extract (PL and GL content, 0.25 ± 0.03 g/100 g DW and 1.31 ± 0.04 g/100 g DW, respectively), may justify the greatest antioxidant performance in this seaweed. The degree of inhibition of ROS was found to be correlated to the amounts of PUFA, the *n*−3 and *n*−6 PUFA ratios in the extracts, and with the GL classes MGDG and DGDG, showing greater antioxidant activities than SGDG [60]. Generally, PUFA and *n*-3 FA have been suggested to hold increased antioxidant potential with regard to other FA [61], and lipid extracts from *S. muticum* are significantly more abundant in these than those of *B. bifurcata*, what might concur to the increased antioxidant activity in extracts from the invasive species. Moreover, it was reported that MGDG containing 20:5, 18:3, and 18:4 [42,53,54,55] and DGDG rich in 20:5 [56,61], which are all FA with increased amounts in *S. muticum*, present biological activity. Synergic effects of the whole lipid compounds contributing to the effective antioxidant outcome cannot be excluded. *Sargassum muticum* lipid extracts are differentiated from those of *B*. *bifurcata* by the specific presence of a huge number of galactolipids, phospholipids, and betaine lipids found in the lipidome of this invasive seaweed, some not found in *B. bifurcata*, which could contribute to its higher antioxidant potential.

Oxidative stress is thought to play a pivotal role in a significant range of diseases, including cardiovascular and neurodegenerative diseases, diabetes, autoimmune processes, and cancer [62]. Moreover, inflammation is being positively correlated with chronic metabolic and inflammatory diseases associated with modern lifestyle [63], and a close relationship between oxidative stress and inflammation is established [64]. All this makes the screening for antioxidant properties a good place to start in terms of bioactivity, while also justifying a constant search for novel compounds with antioxidant and anti-inflammatory properties. This study has justified the prospect of deeper research of the scavenging activity in lipid extract of seaweeds. These lipid extracts can be eventually used as a natural antioxidant in the food industry as well as dietary supplements with antioxidant upside or be used in the cosmetic industry.

## 5. Conclusions

This work meaningfully contributed to the valorization of local natural resources in the brown seaweeds *B. bifurcata* and *S. muticum,* by providing a thorough characterization of their lipidome and highlighting their unique lipidomics features. *Sargassum muticum*, an invasive seaweed of the Portuguese coast, revealed a wide diversity in its polar lipid profile, displaying 13 different classes with 217 lipid species. Either way, either this high lipid variability is correlated to the adaptation to environmental and geographic changes experienced by *S. muticum* while intruding different settlements, or it contributes to the invasive potential of this species. In its turn, *B. bifurcata*, a native seaweed restricted to the environmental conditions particular to a given natural area, revealed a more narrow polar lipid profile, totaling nine different classes with 143 lipid species. Both seaweeds of the Portuguese coast present an interesting PUFA content with interesting nutritional value, namely a high *n*-3 FA content. These features highlight further possible uses in the nutraceutical/supplement food scope. The lipid extracts of the studied seaweeds presented lipid molecular species that have been specifically assigned biological activities, and displayed promising antioxidant properties, especially those of *S. muticum*. This may open new perspectives for the use of these species of algae for the chemical exploration of novel and more effective natural antioxidant compounds representing innovative functional ingredients in foods or as resources for the pharmaceutical and cosmetic industries. Generally, *S. muticum* presented a more varied lipid profile and more enticing results in terms of FA composition and antioxidant activity, which may concur for its further exploitation as a natural resource. The prospect of using an invasive species such as *S. muticum* for further nutritional purposes and as a source of polar lipids with antioxidants activity seems especially attractive, given the great biomass availability and the fact that its use would mean its gradual removal from the invaded habitats.

## Figures and Tables

**Table 1:** Fatty acid profile of *Bifurcaria bifurcata* and *Sargassum muticum* determined by gas chromatography-mass spectrometry (GC–MS) analysis fatty acid methyl esters (FAME) and expressed as relative abundance mean (%) + SD, *n* = 8.

Fatty Acid	*B. bifurcata* (%)	*S. muticum* (%)
14:0	4.04 ± 1.18%	3.00 ± 0.17%
15:0	0.90 ± 0.36%	0.77 ± 0.05%
16:0	30.07 ± 2.78%	24.18 ± 0.48%
16:1	2.84 ± 0.56%	2.34 ± 0.43%
16:2	–	1.65 ± 0.86%
18:0	10.42 ± 7.06%	3.29 ± 0.33%
18:1 n-9	12.12 ± 1.22%	7.91 ± 0.14%
18:2 *n*-6	3.22 ± 0.45%	5.61 ± 0.26%
18:3 *n*-6	0.37 ± 0.26%	0.67 ± 0.09%
18:3 *n*-3	3.84 ± 0.51%	7.07 ± 0.07%
18:4 *n*-3	5.02 ± 0.73%	8.03 ± 0.15%
20:0	0.67 ± 0.18%	0.64 ± 0.12%
20:1	2.23 ± 0.33%	1.56 ± 0.10%
20:2	–	1.46 ± 0.14%
20:3	4.08 ± 1.31% ^(a)^	3.57 ± 0.33% ^(a)^
20:4 *n*-3	1.19 ± 0.20%	0.72 ± 0.09%
20:4 *n*-6	14.28 ± 0.72%	12.53 ± 0.72%
20:5 *n*-3	4.72 ± 0.26%	9.71 ± 0.34%
22:0	–	0.74 ± 0.10%
22:1	–	4.56 ± 1.05%
∑ SFA	46.09 ± 4.30%	32.62 ± 0.92%
∑ MUFA	17.19 ± 1.95%	16.36 ± 0.49%
∑ PUFA	36.72 ± 2.55%	51.02 ± 4.79%
∑ (*n*-3)	14.77 ± 1.28%	25.53 ± 0.49%
∑ (*n*-6)	17.87 ± 0.74%	18.81 ± 0.94%
*n*-6/*n*-3	1.22 ± 0.09	0.74 ± 0.03

(a)With contribution of unknown compound. (–) not detected.

**Table 2:** Polar lipid classes identified by hydrophilic interaction liquid chromatography-high resolution mass spectrometry (HILIC LC–MS) in total lipid extracts of *B. bifurcata* and *S. muticum* with the indication of the total number of lipid species identified in each class and the major lipid species per class.

Lipid Classes	*Bifurcaria bifurcata*		*Sargassum muticum*	
	Lipid Species Number	Major Species	Lipid Species Number	Major Species
**MGDG**	26	MGDG (34:1)	35	MGDG (38:9)
**MGMG**	–	–	10	MGMG (18:4)
**DGDG**	14	DGDG (38:9)	17	DGDG (38:9)
**SQDG**	28	SQDG (34:1)	32	SQDG (34:1)
**LPC**	–	–	3	LPC (16:0)
**PC**	3	PC (30:3)	10	PC (30:3)
**LPE**	–	–	2	LPE (20:4)
**PE**	24	PE (40:8)	34	PE (40:8)
**LPG**	–	–	1	LPG (16:0)
**PG**	10	PG (34:1)	15	PG (34:4)
**PI**	7	PI (38:8)	8	PI (34:2)
**DGTS**	5	DGTS (34:1)	13	DGTS (34:2)
**DGTA**	26	DGTA (36:4)	37	DGTA (36:4)
**Glycolipids**	68	–	94	–
**Phospholipids**	44	–	73	–
**Betaine lipids**	31	–	50	–
Total	143	–	217	–

(–) not detected, MGDG: monogalactosyl diacylglycerol; MGMG: monogalactosyl monoacylglycerol; DGDG: digalactosyl diacylglycerol; SQDG: sulfoquinovosyl diacylglycerol; LPC: lysophosphatidylcholine; PC: phosphatidylcholine; LPE: lysophosphatidylethanolamine; PE: phosphatidylethanolamine; LPG: lysophosphatidylglycerol; PG: phosphatidylglycerol; PI: phosphatidylinositol; DGTS: diacylglyceroltrimethylhomoserine; DGTA: diacylglyceroltrimethyl-β-alanine.

**Table 3:** Antioxidant activity of *B. bifurcata* and *S. muticum* total lipid extracts expressed as inhibitory concentration (IC) values (μg mL^−1^) for ABTS and DPPH assays, and Trolox Equivalents (TE) (µmol g^−1^) for each assay.

Species	ABTS Assay		DPPH Assay	
	IC_50_ (μg mL^−1^)	TE (µmol g^−1^)	IC * (μg mL^−1^)	TE (µmol g^−1^)
***B. bifurcata***	134.19 ± 2.12	100.33 ± 1.57	155.00 ± 3.96	74.89 ± 1.93
***S. muticum***	23.42 ± 0.79	648.35 ± 21.43	188.43 ± 20.55	124.19 ± 14.39

* IC_25_ for *B. bifurcata* and IC_50_ for *S. muticum*.

## Supplementary Materials

The following are available online at https://www.mdpi.com/2076-3921/9/7/642/s1; **Figure S1.** LC−MS/MS spectra of SQDG (34:1), **Figure S2**. LC−MS/MS spectra of [M + NH4]+ ions of MGDG (36:2), **Figure S3.** LC−MS/MS spectrum of [M + NH_4_]^+^ ion of DGDG (32:2), **Figure S4.** LC−MS/MS spectra of [M + H]^+^ ion at *m/z* 736.6 of DGTS (34:2), **Figure S5.** LC−MS/MS spectra of [M − H]^−^ ions of PG (34:4) at m/z 741.5, **Figure S6.** LC-MS/MS spectra of the [M + H]^+^ ion of LPE (20:4) at *m/z* 502.3, **Table S1**. Molecular species identified by HILIC-MS in B. bifurcate, **Table S2.** Molecular species identified by HILIC-MS and MS/MS in S. muticum, **Table S3** List of common and unique lipid species in the lipidome of B. bifurcata and S. muticum.
