# Supplementary material for: Unraveling the Lipidome and Antioxidant Activity of Native Bifurcaria bifurcata and Invasive Sargassum muticum Seaweeds: A Lipid Perspective on How Systemic Intrusion May Present an Opportunity
Source: Antioxidants (Basel). 2020 Jul 21;9(7):642. doi: 10.3390/antiox9070642 (PMC7420230; doi:10.3390/antiox9070642)
Supplement: Supplementary file 1 [file antioxidants-09-00642-s001.pdf]

Supplementary material

Unraveling the lipidome and antioxidant activity of native *Bifurcaria bifurcata* and invasive *Sargassum muticum* seaweeds: A lipid perspective on how systemic intrusion may present an opportunity

Fábio Santos [1], João P. Monteiro [1,2], Daniela Duarte [1], Tânia Melo [1,2], Diana Lopes [1,2], Elisabete da Costa[1,2], and M. Rosário Domingues[1,2,*]

[1] Mass Spectrometry Centre, LAQV-REQUIMTE, Department of Chemistry, University of Aveiro, Santiago University Campus, 3810-193 Aveiro, Portugal; fabiofs379@gmail.com (F.S.), jpspmonteiro@yahoo.com (J.M.), danieladuarte98@ua.pt (D.D.), taniamelo@ua.pt (T.M.), dianasalzedaslopes@ua.pt (D.L.), elisabetecosta@ua.pt (E.d.C.), mrd@ua.pt (M.R.D.)

[2] CESAM - Centre for Environmental and Marine Studies, Department of Chemistry, University of Aveiro, Santiago University Campus, 3810-193 Aveiro, Portugal

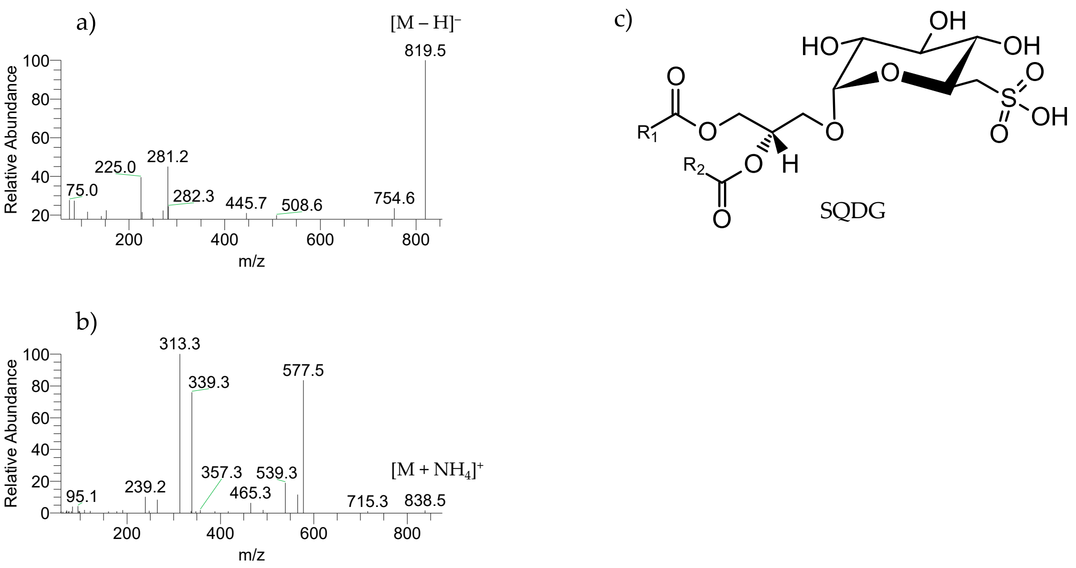

Figure S1. LC–MS/MS spectra of SQDG (34:1), namely of the $[M - H]^-$ ion at *m/z* 819.5 (a) and $[M + NH_4]^+$ ion at *m/z* 838.5 (b). The SQDG (34:1), was identified as SQDG (16:0/18:1) species. Typical fragmentation of SQDG species observed in LC-MS/MS spectrum of $[M - H]^-$ ions at *m/z* 819.5 showed the characteristic ion at *m/z* 225.0, corresponding to the dehydrosulfoglycosyl anion ($[C_6H_9O_7S]^-$) of the polar head group that confirms the sulfoglycolipid class (a). The neutral loss of 261 Da, corresponding to the loss of sulfoglycosyl group ($C_6H_{12}O_8S$) and ammonia (NH_3) observed in LC-MS/MS spectrum of $[M + NH_4]^+$ ion, confirms the sulfonoglycolipid identity. The fatty acyl chains can be confirmed in positive mode due to the presence of the acylium ion of fatty acyl chain plus 74 ($[RCO + 74]^+$ ions). These ions can be seen at *m/z* 313.3 and *m/z* 339.3 in the LC-MS/MS spectrum of $[M + NH_4]^+$ ion of SQDG (16:0/18:1), corresponding to 16:0 and 18:1 fatty acids, confirming the SQDG (16:0/18:1) species (c). The representative structure of SQDG is depicted (c).

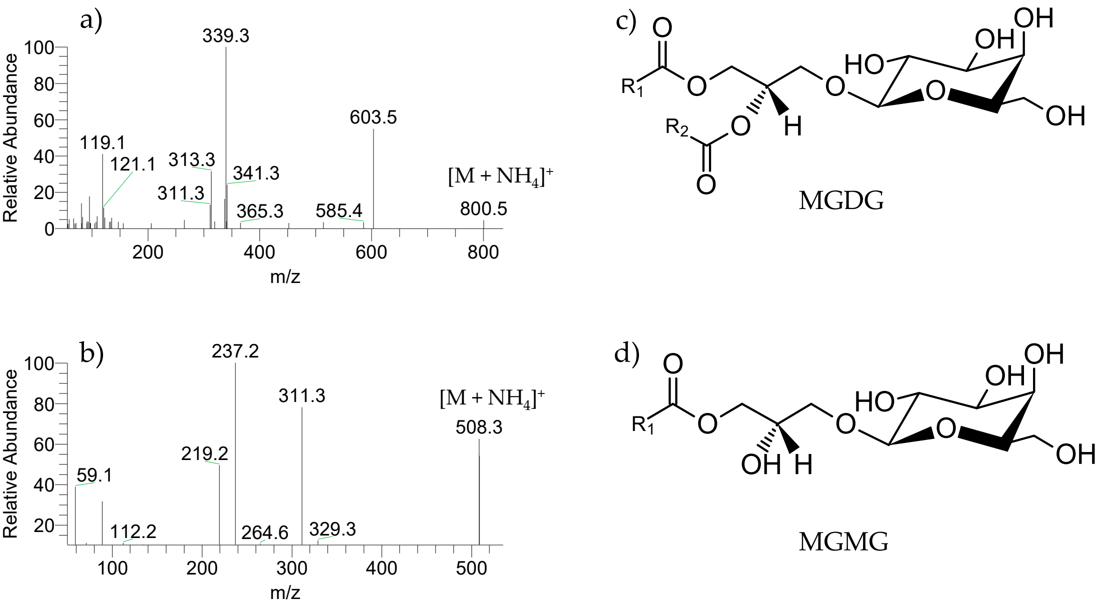

Figure S2. LC–MS/MS spectra of [M + NH_4]$^+$ ions of MGDG (36:2), at *m/z* 800.5 corresponding to MGDG (16:0/20:2), (18:0/18:2), (18:1/18:1) and (16:1/20:1) species, (a) and of [M + NH_4]$^+$ ion of MGMG (16:1) at *m/z* 508.3 (b). The LC-MS/MS spectrum of the [M + NH_4]$^+$ ion of MGDG (36:2) at *m/z* 800.5 (a) showed a typical combined loss of NH_3 plus loss of galactosyl unit (-197 Da), a typical fragmentation of MGDG species with formation of the product ion at *m/z* 603.5 [1,2]. The product ions [RCO + 74]$^+$ seen at *m/z* 311.3, *m/z* 313.3, *m/z* 339.3, *m/z* 341.3 and *m/z* 365.3, corresponding to the fatty acids 16:1, 16:0, 18:1, 18:0 and 20:2, respectively, allowed to pinpoint fatty acyl composition and to propose the contribution of the lipid molecular species MGDG (16:0/20:2), (18:1/18:1), (18:0/18:2) and (16:1/20:1) for the MGDG (36:2) (Table S1). The LC-MS/MS spectrum of [M+NH_4]$^+$ of MGMG (16:1) at *m/z* 508.3 (b) showed the typical neutral loss of 197 Da, due to the combined loss of NH_3 and galactosyl residue (-179 Da), with the formation of the product ion at *m/z* 311.3. For the MGMG class, the fatty acyl chain composition can be observed as [RCO]$^+$ and [RCO + 74]$^+$ product ions, seen at *m/z* 237.2 and *m/z* 311.3, respectively, which correspond to the fatty acid 16:1 confirming the presence of the MGMG (16:1) (b). The representative structure of MGDG (c) and MGMG (d) are depicted in the figure.

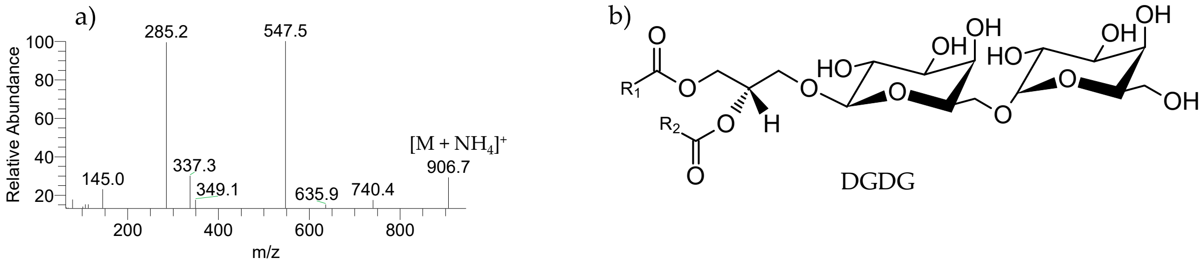

Figure S3. LC–MS/MS spectrum of [M + NH_4]$^+$ ion of DGDG (32:2) at *m/z* 906.7 corresponding to the DGDG (14:0/18:2) species (a). The typical fragmentation of this class of GL, namely neutral loss of the carbohydrate moiety (loss of 180 + 162 Da) combined with loss of NH_3 (-17 Da) is seen as a total neutral loss of (-359 Da) with formation of the product ion at *m/z* 547.5. The [RCO + 74]$^+$ product ions allow to confirm the fatty acyl composition and are seen at *m/z* 285.2 and *m/z* 337.3, corresponding to the fatty acids 14:0 and 18:2, respectively, confirming the presence of the DGDG (14:0/18:2). The representative structure of DGDG is depicted in the figure.

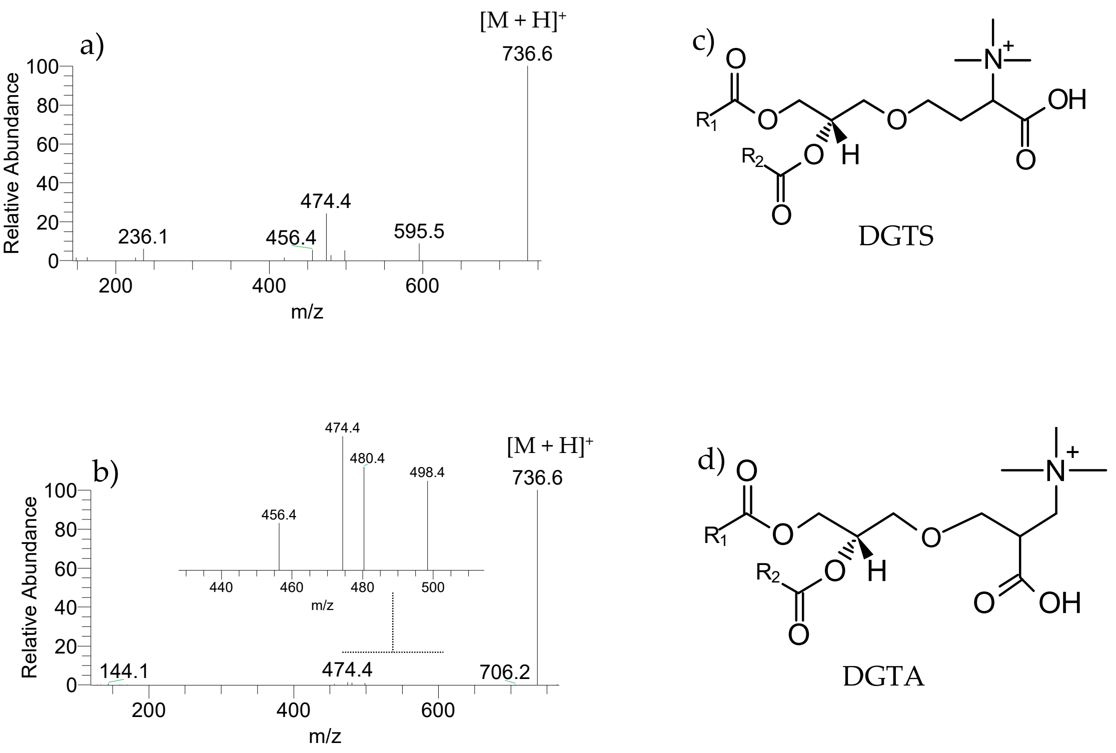

Figure S4. LC–MS/MS spectra of $[M + H]^+$ ion at *m/z* 736.6 of DGTS (34:2), corresponding to the DGTS (16:0/18:2) species (a) and the DGTA (34:2), namely DGTA (16:0/18:2) species, at *m/z* 736.6 (b). These two isomeric betaine classes eluted at different retention times: DGTS at RT = 4.2 and DGTA at RT = 9.4. LC-MS/MS spectra of DGTS (34:2) (a) and DGTA (34:2) (b) show the typical reported product ions of these classes at *m/z* 144.1, corresponding to loss of both fatty acyl groups as keto derivatives (R_1CO + R_2CO) plus glycerol, and at *m/z* 236.1 corresponding to the loss of both fatty acyl chains as keto derivatives (R_1CO + R_2CO) [3–5]. These characteristic product ions at *m/z* 144.1 and *m/z* 236.1 can be used to conduct the qualitative characterization of DGTS and DGTA classes as positive $[M + H]^+$ ions, but it can't be used to differentiate DGTS from DGTA, since both ions can be present in DGTA and DGTS spectra [6]. In these cases, the DGTA and DGTS differentiation can only be made by the specific retention time of each class, as mentioned before [6]. The fatty acyl composition can be assigned by the losses of fatty acyl chains as acid (-RCOOH) and ketene (-R=C=O) derivatives. In the spectrum of DGTS (16:0/18:2) (a), the ion at *m/z* 474.4 corresponds to the loss of 18:2 fatty acyl chain as keto derivative (-262 Da) and the at *m/z* 456.4 corresponds to 18:2 as RCOOH (-280 Da), confirming the presence of the fatty acid 18:2. In the LC-MS/MS spectrum of DGTA (16:0/18:2) (b), the ions at *m/z* 498.4 and 474.4 corresponding to the loss of fatty acyl chains as keto derivatives (-238 and -262 Da), match to 16:0 and 18:2 fatty acids, respectively. Moreover, the ions at *m/z* 480.4 and 456.4 confirmed the presence of the fatty acids 16:0 and 18:2, respectively, since they correspond to the loss of RCOOH (-256 and -280 Da). The representative structures of DGTS (c) and DGTA (d) are depicted in the figure.

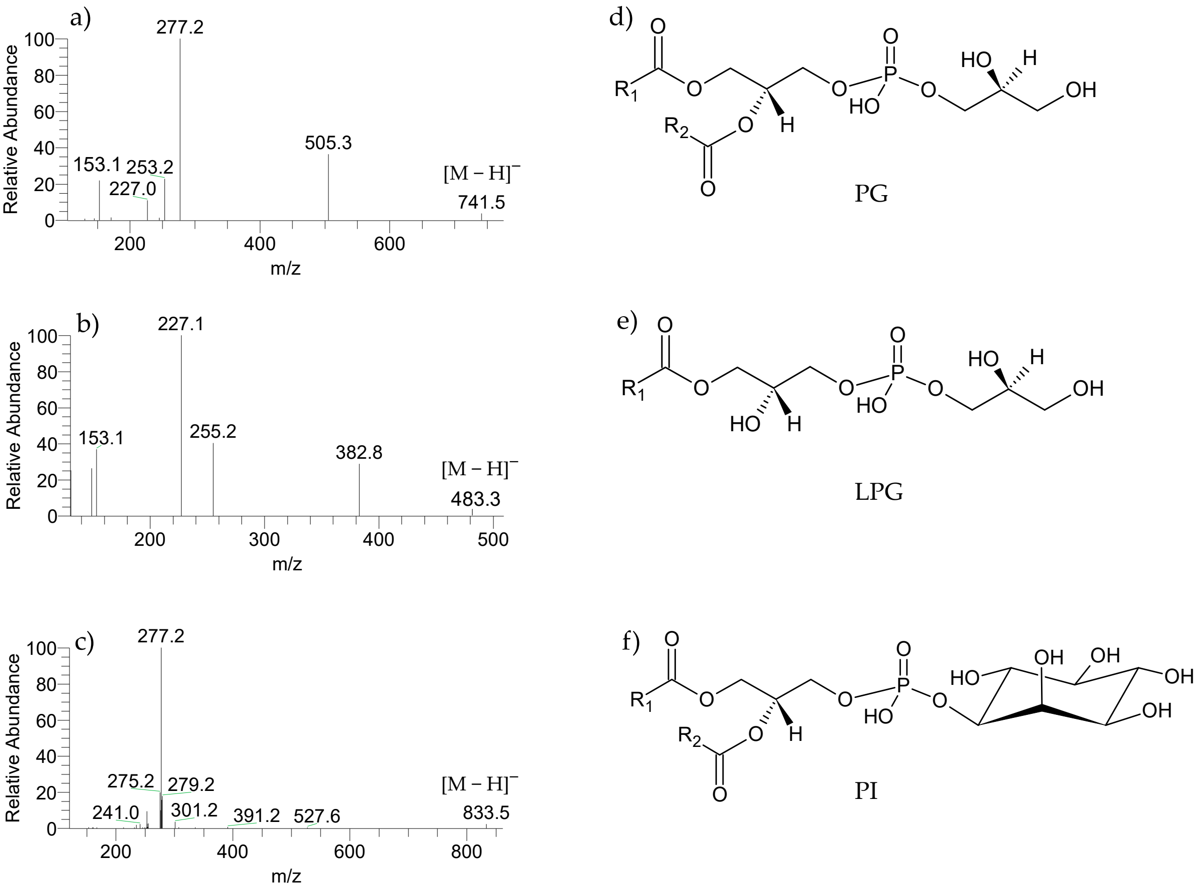

Figure S5. LC–MS/MS spectra of [M − H]⁻ ions of PG (34:4) at *m/z* 741.5, corresponding to the PG (18:3/16:1) species (a), LPG (16:0) at *m/z* 483.3 (b), and PI (34:2) at *m/z* 833.5 corresponding to the PI (18:2/16:0) species, (c). PG (a) and LPG (b) species were identified by the presence of the product ion at *m/z* 153.1, corresponding to glycerol phosphate minus one water molecule, and *m/z* 227.1 corresponding to $[C_6H_{12}O_7P]^-$ anion corresponding to glycerophosphate glycerol minus one water molecule [3]. The PI class (c) was identified in the MS/MS spectra through the characteristic product ion at *m/z* 241.0 that corresponds to the inositol-1,2-cyclic phosphate anion ($[C_6H_{10}O_5PO_3]^-$) of the polar head of PI [1].The carboxylate anions (R_1COO^- and R_2COO^-) allowed the identification of fatty acyl chains in PG, its lyso form, and PI [7]. The representative structures of PG (d), LPG (e), and PI (f) are depicted in the figure.

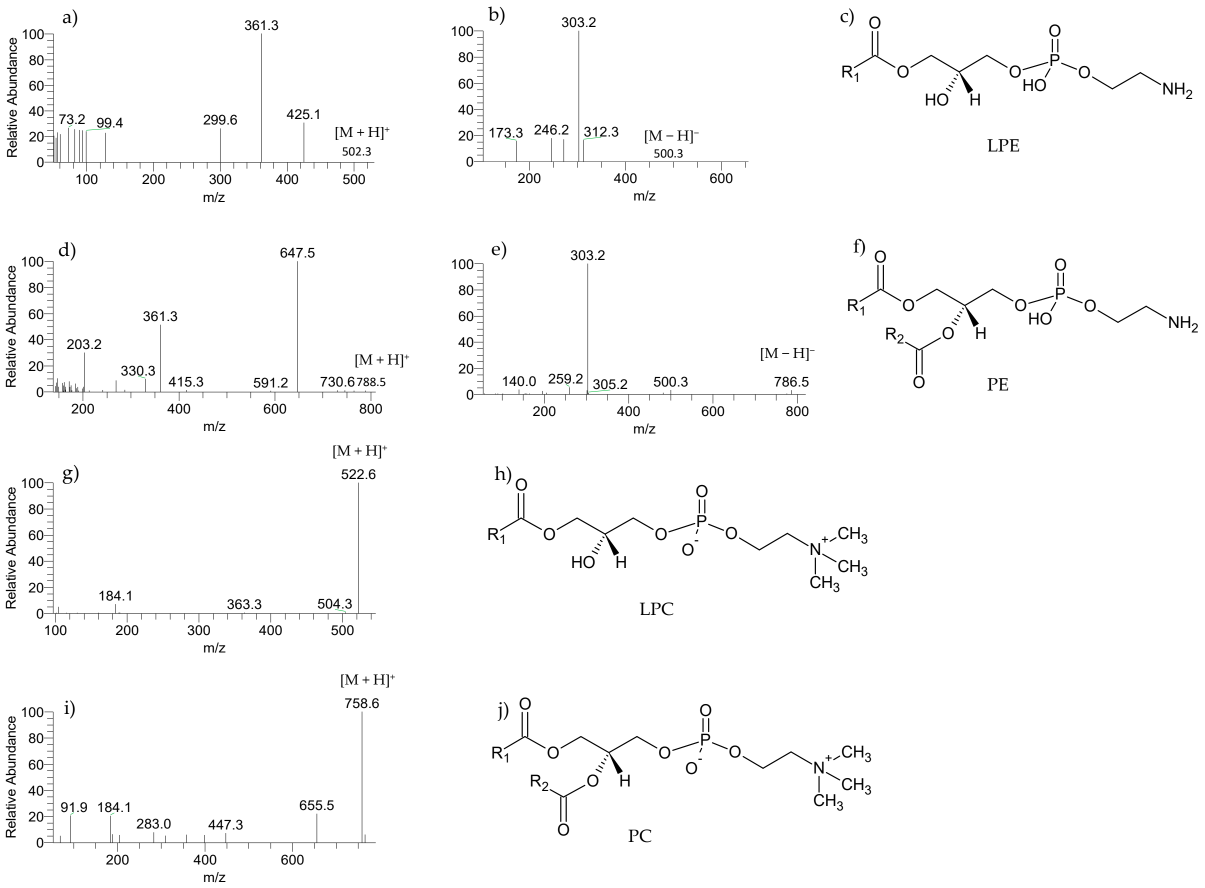

Figure S6. LC-MS/MS spectra of the [M + H]$^+$ ion of LPE (20:4) at *m/z* 502.3 (a), [M − H]$^-$ ion of LPE (20:4) at *m/z* 500.3 (b); [M + H]$^+$ ion of PE (40:8) at *m/z* 788.5, corresponding to the PE (20:4/20:4) and PE (20:3/20:5) species (d); [M − H]$^-$ ion of PE (40:8) at *m/z* 786.5 corresponding to the PE (20:4/20:4) and PE (20:3/20:5) species (e); [M + H]$^+$ ion at *m/z* 522.6 of LPC (18:1) (g); and [M+H]$^+$ ion at *m/z* 758.6 of PC (34:2) (i). Typical loss of 141 Da was observed in the LC-MS/MS spectra of [M + H]$^+$ ions of LPE (a) and PE (d), while the carboxylate anions (RCOO$^-$) of the fatty acyl chains were identified in the LC-MS/MS spectra of [M − H]$^-$ ions of LPE (b) and PE (e). The LC-MS/MS spectra of [M + H]$^+$ ions of LPC (g) and PC (i) showed the typical product ion of the polar head at *m/z* 184.0. The representative structures of LPE (c), PE (f), LPC (h), and PC (j) are depicted in the figure.

Tandem mass spectrometry analysis was based on the following literature:

1. Lopes, D.; Moreira, A. S. P.; Rey, F.; da Costa, E.; Melo, T.; Maciel, E.; Rego, A.; Abreu, M. H.; Domingues, P.; Calado, R.; Lillebø, A. I.; Rosário Domingues, M. Lipidomic signature of the green macroalgae *Ulva rigida* farmed in a sustainable integrated multi-trophic aquaculture. *J. Appl. Phycol.* **2018**.
2. Da Costa, E.; Domingues, P.; Melo, T.; Coelho, E.; Pereira, R.; Calado, R.; Abreu, M. H.; Domingues, M. R. Lipidomic signatures reveal seasonal shifts on the relative abundance of high-valued lipids from the brown algae *Fucus vesiculosus*. *Mar. Drugs* **2019**.
3. Melo, T.; Alves, E.; Azevedo, V.; Martins, A. S.; Neves, B.; Domingues, P.; Calado, R.; Abreu, M. H.; Domingues, M. R. Lipidomics as a new approach for the bioprospecting of marine macroalgae — Unraveling the polar lipid and fatty acid composition of *Chondrus crispus*. *Algal Res.* **2015**.
4. Da Costa, E.; Melo, T.; Moreira, A. S. P. A.; Bernardo, C.; Helguero, L.; Ferreira, I.; Cruz, M. T. M.; Rego, A. M. A.; Domingues, P.; Calado, R.; Abreu, M. M. H.; Domingues, M. Valorization of lipids from *Gracilaria* sp. through lipidomics and decoding of antiproliferative and anti-Inflammatory Activity. *Mar. Drugs* **2017**.
5. Roche, S. A.; Leblond, J. D. Betaine lipids in chlorarachniophytes. *Phycol. Res.* **2010**.
6. Li, Y.; Lou, Y.; Mu, T.; Xu, J.; Zhou, C.; Yan, X. Simultaneous structural identification of diacylglyceryl-N-trimethylhomoserine (DGTS) and diacylglycerylhydroxymethyl-N,N,N-trimethyl-β-alanine (DGTA) in microalgae using dual Li+/H+ adduct ion mode by ultra-performance liquid chromatography/quadrupole ti. *Rapid Commun. Mass Spectrom.* **2017**.
7. Murphy, R. C. *Tandem Mass Spectrometry of Lipids*; Ron M A Heeren Mitsutoshi Setou, R. C. M., Ed.; Royal Soci.; TGhomas Graham House, Science Park, Milton Road, 2014; ISBN 9781849738279.

Table S1. Molecular species identified by HILIC-MS in *B. bifurcata*. Identification of different polar lipid classes and fatty acyl composition was confirmed by the analysis of the LC-MS/MS spectra of each ion. C represents the total number of carbon atoms and N the total number of double bonds on the fatty acyl chains. The most abundant species are marked in bold.

Lipid species (C:N)	Observed m/z	Delta (ppm)	Retention time	Fatty acyl chains (C:N)	Formula
			**MGDG identified as $[M + NH_4]^+$**		
MGDG(30:4)	712.5000	0.0000	2.05	(14:0/16:4)	C39H70NO10
MGDG(30:1)	718.5477	1.8092	2.16	(14:0/16:1)	C39H76NO10
MGDG(32:4)	740.5307	0.0000	2.08	(16:0/16:4) and (14:1/18:3)	C41H74NO10
MGDG(32:3)	742.5487	3.0974	2.32	(18:3/14:0) and (16:1/16:2)	C41H76NO10
MGDG(32:2)	744.5618	-1.0745	2.13	(16:1/16:1) and (18:2/14:0)	C41H78NO10
MGDG(32:1)	746.5775	-0.2679	2.13	(16:0/16:1) and (18:1/14:0)	C41H80NO10
MGDG(34:4)	768.5627	0.1301	2.08	(18:4/16:0), (18:3/16:1) and (14:0/20:4)	C43H78NO10
MGDG(34:3)	770.5771	-1.4586	2.13	(16:0/18:3) and (18:2/16:1)	C43H80NO10
MGDG(34:2)	772.5932	-0.1294	2.13	(16:0/18:2)	C43H82NO10
**MGDG(34:1)**	**774.6092**	**0.2582**	**2.16**	**(16:0/18:1)**	**C43H84NO10**
MGDG(36:8)	788.5308	-0.596	2.21	(18:4/18:4) and (20:4/16:4)	C45H74NO10
MGDG(36:7)	790.5469	0.0000	2.16	(18:3/18:4)	C45H76NO10
MGDG(36:6)	792.5616	-1.1356	2.18	(18:3/18:3) and (18:4/18:2)	C45H78NO10
MGDG(36:5)	794.5788	0.7551	2.13	(18:3/18:2), (20:4/16:1) and (20:5/16:0)	C45H80NO10
MGDG(36:4)	796.5942	1.1298	2.16	(18:3/18:1), (18:2/18:2) and (20:4/16:0)	C45H82NO10
MGDG(36:3)	798.6082	-1.6529	2.16	(16:1/20:2), (18:2/18:1) and (16:0/20:3)	C45H84NO10
MGDG(36:2)	800.6249	-0.3422	2.18	(18:1/18:1) and (16:0/20:2)	C45H86NO10
MGDG(38:10)	812.5319	0.7754	2.02	*	C47H74NO10
MGDG(38:9)	814.5463	-0.7661	2.13	(20:5/18:4)	C47H76NO10
MGDG(38:8)	816.5617	-1.0654	2.13	(20:4/18:4) and (20:5/18:3)	C47H78NO10
MGDG(38:7)	818.5761	-2.5947	2.18	(18:3/20:4)	C47H80NO10
MGDG(38:6)	820.5934	-0.6093	2.16	(18:1/20:5)	C47H82NO10
MGDG(38:5)	822.6093	-0.2674	2.08	(18:1/20:4), (20:3/18:2), (18:3/20:2) and (18:4/20:1)	C47H84NO10
MGDG(40:10)	840.5645	2.2961	1.77	(20:5/20:5)	C49H78NO10
MGDG(40:9)	842.5782	0.0000	2.13	(20:5/20:4)	C49H80NO10
MGDG(40:8)	844.5933	-0.6749	2.13	(20:4/20:4)	C49H82NO10
			**DGDG identified as $[M + NH_4]^+$**		
DGDG(28:0)	854.582	-2.4573	2.74	*	C43H84O15N
DGDG(32:3)	904.5995	-0.2211	2.32	(14:0/18:3)	C47H86O15N
DGDG(32:2)	906.6154	0.0000	2.35	(18:2/14:0) and (16:1/16:1)	C47H88O15N
DGDG(32:1)	908.6310	0.0000	2.33	(16:0/16:1) and (14:0/18:1)	C47H90O15N
DGDG(34:4)	930.6154	0.0000	2.30	(18:3/16:1) and (16:0/18:4)	C49H88O15N
DGDG(34:3)	932.6308	-0.2144	2.33	(18:3/16:0)	C49H90O15N
DGDG(34:2)	934.6457	-1.0699	2.44	(16:0/18:2) and (18:1/16:1)	C49H92O15N
DGDG(34:1)	936.6619	-0.427	2.32	(16:0/18:1)	C49H94O15N

DGDG(36:7)	952.6001	0.4199	2.39	(18:4/18:3)	C51H86O15N
DGDG(36:6)	954.6152	-0.2095	2.32	(18:3/18:3) and (16:1/20:5)	C51H88O15N
DGDG(36:5)	956.6299	-1.1499	2.33	(20:5/16:0), (18:3/18:2) and (16:1/20:4)	C51H90O15N
DGDG(36:4)	958.6467	0.0000	2.39	*	C51H92O15N
**DGDG(38:9)**	**976.5997**	**0.0000**	**2.46**	**(18:4/20:5)**	**C53H86O15N**
DGDG(38:8)	978.6155	0.1022	2.45	*	C53H88O15N
			**SQDG identified as [M − H]⁻**		
SQDG(30:1)	763.4667	0.0956	1.69	(14:0/16:1)	C39H71O12S
SQDG(30:0)	765.4823	0.0300	1.82	(16:0/14:0)	C39H73O12S
SQDG(32:3)	787.4667	0.0927	1.86	(14:0/18:3)	C41H71O12S
SQDG(32:2)	789.4823	0.0291	1.75	(16:0/16:1)	C41H73O12S
SQDG(32:1)	791.4988	1.1030	1.80	(16:1/16:0) and (18:1/14:0)	C41H75O12S
SQDG(32:0)	793.5135	-0.0958	1.69	*	C41H77O12S
SQDG(34:5)	811.4666	-0.0333	1.72	*	C43H71O12S
SQDG(34:4)	813.4839	1.9951	1.75	(18:4/16:0)	C43H73O12S
SQDG(34:3)	815.4996	2.0515	1.82	(18:3/16:0) and (18:2/16:1)	C43H75O12S
SQDG(34:2)	817.5143	0.8856	1.72	(18:1/16:1)	C43H77O12S
**SQDG(34:1)**	**819.5295**	**0.3331**	**1.75**	**(14:0/20:1), (16:0/18:1) and (16:1/18:0)**	**C43H79O12S**
SQDG(34:0)	821.5449	0.0280	1.8	(16:0/18:0)	C43H81O12S
SQDG(36:7)	835.4666	-0.0323	1.72	**	C45H71O12S
SQDG(36:6)	837.4823	0.0275	1.75	**	C45H73O12S
SQDG(36:5)	839.4978	-0.1513	1.72	(16:0/20:5)	C45H75O12S
SQDG(36:4)	841.5137	0.1474	1.80	(16:0/20:4)	C45H77O12S
SQDG(36:3)	843.5291	-0.1506	1.69	**	C45H79O12S
SQDG(36:2)	845.5445	-0.4459	1.75	(18:1/18:1)	C45H81O12S
SQDG(38:9)	859.4666	-0.0314	1.82	*	C47H71O12S
SQDG(38:8)	861.4823	0.0267	1.87	**	C47H73O12S
SQDG(38:7)	863.4979	-0.0313	1.82	(20:5/18:2)	C47H75O12S
SQDG(38:6)	865.5136	0.0277	1.75	*	C47H77O12S
SQDG(38:5)	867.5305	1.4674	1.82	(18:1/20:4)	C47H79O12S
SQDG(38:4)	869.5455	0.7165	2.18	**	C47H81O12S
SQDG(38:0)	877.6075	0.0262	1.75	**	C47H89O12S
SQDG(40:8)	889.5135	-0.0854	1.86	**	C49H77O12S
SQDG(40:7)	891.5293	0.0819	1.87	*	C49H79O12S
SQDG(40:0)	905.6387	-0.0850	1.82	**	C49H93O12S
			**PC identified as [M + H]⁺**		
**PC(30:3)**	**700.4913**	**-0.6167**	**12.15**	*	**C38H71NO8P**
PC(34:2)	758.5700	0.0237	11.88	**	C42H81NO8P
PC(38:6)	806.5700	0.0223	11.28	*	C46H81NO8P
			**PE identified as [M + H]⁺**		
PE(30:3)	658.4426	-3.3139	4.57	**	C35H65NO8P
PE(34:4)	712.4924	0.9376	4.05	(14:0/20:4)	C39H71NO8P
PE(34:2)	716.5234	0.5136	4.19	*	C39H75NO8P

PE(34:1)	718.5387	0.0251	4.05	(14:0/20:1)	C39H77NO8P
PE(36:5)	738.5078	0.5660	4.10	(16:1/20:4) and (16:0/20:5)	C41H73O8NP
PE(36:4)	740.5226	-0.5834	4.05	**	C41H75NO8P
PE(36:3)	742.5383	-0.5145	4.20	**	C41H77NO8P
PE(36:2)	744.5557	1.8387	4.09	(16:1/20:1)	C41H79O8NP
PE(38:8)	760.4889	-3.7239	4.01	**	C43H71NO8P
PE(38:7)	762.5059	-1.9436	3.99	**	C43H73NO8P
PE(38:6)	764.5227	-0.4343	4.05	(18:1/20:5) and (20:4/18:2)	C43H75O8NP
PE(38:5)	766.5369	-2.3247	4.05	(20:4/18:1) and (20:5/18:0)	C43H77NO8P
PE(38:4)	768.553	-1.7318	4.05	**	C43H79O8NP
PE(40:10)	784.4918	0.0867	4.05	*	C45H71NO8P
PE(40:9)	786.5071	-0.3585	3.92	(20:4/20:5)	C45H73O8NP
**PE(40:8)**	**788.5228**	**-0.2942**	**3.92**	**(20:4/20:4)**	**C45H75O8NP**
PE(40:7)	790.5389	0.2758	3.78	*	C45H77NO8P
PE(40:6)	792.5543	-0.0391	3.92	(20:4/20:2) and (20:3/20:3)	C45H79O8NP
PE(40:5)	794.5700	0.0227	3.92	(16:0/24:5)	C45H81O8NP
PE(40:4)	796.5869	1.5918	3.86	(20:0/20:4)	C45H83NO8P
PE(42:11)	810.5045	-3.5558	3.85	**	C47H73NO8P
PE(42:5)	822.6013	0.0219	3.92	(20:4/22:1) and (20:5/22:0)	C47H85O8NP
PE(42:4)	824.6169	-0.0388	3.92	(22:0/20:4)	C47H87O8NP
PE(44:4)	852.6482	-0.0375	3.92	(20:4/24:0)	C49H91NO8P
**PG identified as [M – H]$^-$**					
PG(32:1)	719.4863	0.0000	1.97	(16:1/16:0) and (18:1/14:0)	C38H72O10P
PG(34:4)	741.4706	-0.1349	1.97	(14:0/20:4) and (16:1/18:3)	C40H70O10P
PG(34:3)	743.4863	0.0000	1.97	(18:3/16:0) and (16:1/18:2)	C40H72O10P
PG(34:2)	745.5032	1.6097	1.92	(14:0/20:2)	C40H74O10P
**PG(34:1)**	**747.5201**	**3.3444**	**1.95**	**(16:0/18:1) and (14:0/20:1)**	**C40H76O10P**
PG(34:0)	749.5336	0.4002	1.95	*	C40H78O10P
PG(36:5)	767.4863	0.0000	1.86	*	C42H72O10P
PG(36:4)	769.502	0.0000	1.92	(20:4/16:0), (18:3/18:1) and (18:2/18:2)	C42H74O10P
PG(36:3)	771.5176	0.0000	1.98	(16:1/20:2), (16:0/20:3) and (18:2/18:1)	C42H76O10P
PG(36:2)	773.5333	0.0000	1.92	(18:1/18:1) and (16:0/20:2)	C42H78O10P
**PI identified as [M – H]$^-$**					
PI(30:2)	777.4554	0.0000	1.62	*	C39H70O13P
PI(34:1)	835.5337	0.0000	1.97	(18:1/16:0)	C43H80O13P
PI(36:8)	849.4554	-0.0094	1.82	*	C45H70O13P
**PI(38:8)**	**877.4878**	**1.2536**	**1.75**	*****	**C47H74O13P**
PI(40:6)	909.5528	3.8481	1.82	*	C49H82O13P
PI(40:5)	911.5664	1.5358	1.87	*	C49H84O13P
PI(46:3)	999.6912	1.0423	2.03	*	C55H100O13P
**DGTS identified as [M + H]$^+$**					
DGTS(32:1)	710.5935	0.0000	4.10	*	C42H80O7N
DGTS(32:2)	708.5779	0.1411	4.10	*	C42H78O7N

DGTS(34:2)	736.6097	0.8145	4.16	*	$C_{44}H_{82}O_7N$
**DGTS(34:1)**	**738.6244**	**-0.5415**	**4.36**	*	**$C_{44}H_{84}O_7N$**
DGTS(36:4)	760.6091	0.00000	4.37	*	$C_{46}H_{82}O_7N$
**DGTA identified as $[M + H]^+$**					
DGTA(30:1)	682.5622	0.0000	10.14	*	$C_{40}H_{76}O_7N$
DGTA(32:4)	704.5465	0.0000	9.46	**	$C_{42}H_{74}O_7N$
DGTA(32:3)	706.5615	-0.9907	9.87	*	$C_{42}H_{76}O_7N$
DGTA(32:2)	708.5782	0.5645	9.66	*	$C_{42}H_{78}O_7N$
DGTA(32:1)	710.5933	-0.2815	9.73	*	$C_{42}H_{80}O_7N$
DGTA(34:5)	730.5619	-0.4106	9.76	**	$C_{44}H_{76}O_7N$
DGTA(34:4)	732.5782	0.5460	9.87	(14:0/20:4)	$C_{44}H_{78}O_7N$
DGTA(34:3)	734.5913	-2.9949	9.87	*	$C_{44}H_{80}O_7N$
DGTA(34:2)	736.6100	1.2218	9.24	(18:2/16:0)	$C_{44}H_{82}O_7N$
DGTA(34:1)	738.6242	-0.8123	9.87	(16:1/18:0) and (16:0/18:1)	$C_{44}H_{84}O_7N$
DGTA(36:7)	754.5622	0.0000	9.67	**	$C_{46}H_{76}O_7N$
DGTA(36:6)	756.5763	-1.9826	9.87	**	$C_{46}H_{78}O_7N$
DGTA(36:5)	758.5927	-1.0546	9.87	(20:5/16:0) and (20:4/16:1)	$C_{46}H_{80}O_7N$
**DGTA(36:4)**	**760.6080**	**-1.4462**	**9.87**	**(16:0/20:4) and (18:0/18:4)**	**$C_{46}H_{82}O_7N$**
DGTA(36:3)	762.6229	-2.4914	10.14	(16:0/20:3)	$C_{46}H_{84}O_7N$
DGTA(36:2)	764.6387	-2.2233	9.87	*	$C_{46}H_{86}O_7N$
DGTA(38:8)	780.5778	0.0000	9.19	**	$C_{48}H_{78}O_7N$
DGTA(38:7)	782.5929	-0.7667	9.00	**	$C_{48}H_{80}O_7N$
DGTA(38:6)	784.6082	-1.1471	9.87	(18:2/20:4)	$C_{48}H_{82}O_7N$
DGTA(38:5)	786.6231	-2.1611	9.42	(18:1/20:4)	$C_{48}H_{84}O_7N$
DGTA(40:9)	806.5935	0.0000	9.00	(20:5/20:4)	$C_{50}H_{80}O_7N$
DGTA(40:8)	808.6087	-0.4947	9.00	(20:4/20:4)	$C_{50}H_{82}O_7N$
DGTA(40:7)	810.6225	-2.8373	9.00	(20:3/20:4)	$C_{50}H_{84}O_7N$
DGTA(40:6)	812.6387	-2.0919	9.81	*	$C_{50}H_{86}O_7N$
DGTA(42:11)	830.5902	-3.9478	9.00	**, #	$C_{52}H_{80}O_7N$
DGTA(42:8)	836.6403	-0.1542	9.05	*	$C_{52}H_{86}O_7N$

*: Molecular species identified by retention time and mass accuracy;

**: Molecular species identified by retention time, mass accuracy, and polar head product ion;

#: Contribution of sodiated adduct ion

Table S2. Molecular species identified by HILIC-MS and MS/MS in *S. muticum*. The identification of different polar lipid classes and fatty acyl composition was confirmed by mass accuracy, retention time and the analysis of the LC-MS/MS spectra of each ion. C represents the total number of carbon atoms and N the total number of double bonds on the fatty acyl chains. The most abundant species are marked in bold.

Lipid species (C:N)	Observed m/z	Delta (ppm)	Retention time	Fatty acyl chains (C:N)	Formula
			**MGDG identified as $[M + NH_4]^+$**		
MGDG(30:1)	718.5464	0.0000	2.40	(14:1/16:0) and (16:1/14:0)	C39H76NO10
MGDG(30:0)	720.5616	-1.2490	2.31	(16:0/14:0)	C39H78NO10
MGDG(32:8)	732.4683	-0.5461	2.14	*	C41H66NO10
MGDG(32:5)	738.5156	-0.0325	2.25	*	C41H72NO10
MGDG(32:4)	740.5307	0.0000	2.22	(14:0/18:4) and (16:1/16:3)	C41H74NO10
MGDG(32:3)	742.5463	-0.1347	2.31	(18:3/14:0), (18:2/14:1) and (16:0/16:3)	C41H76NO10
MGDG(32:2)	744.562	-0.8058	2.31	(16:1/16:1), (16:0/16:2) and (18:2/14:0)	C41H78NO10
MGDG(32:1)	746.5775	-0.2679	2.2	(18:1/14:0) and (16:0/16:1)	C41H80NO10
MGDG(34:7)	762.5163	0.8865	1.87	(16:3/18:4)	C43H72NO10
MGDG(34:6)	764.5314	0.1661	2.04	(18:4/16:2) and (18:3/16:3)	C43H74NO10
MGDG(34:5)	766.5469	-0.0313	2.20	(16:1/18:4), (16:2/18:3) and (16:3/18:2)	C43H76NO10
MGDG(34:4)	768.563	0.5205	2.31	(16:1/18:3)	C43H78NO10
MGDG(34:3)	770.5781	-0.1609	2.20	(18:3/16:0) and (16:1/18:2)	C43H80NO10
MGDG(34:2)	772.5933	0.0000	2.20	(16:0/18:2) and (18:1/16:1)	C43H82NO10
MGDG(34:1)	774.6086	-0.5164	2.20	(18:1/16:0)	C43H84NO10
MGDG(36:9)	786.5158	0.2543	2.32	(20:5/16:4)	C45H72NO10
MGDG(36:8)	788.5308	-0.5960	2.20	(18:4/18:4)	C45H74NO10
MGDG(36:7)	790.5465	-0.5060	2.20	(18:3/18:4)	C45H76NO10
MGDG(36:6)	792.5625	0.0000	2.19	(20:5/16:1) and (18:3/18:3)	C45H78NO10
MGDG(36:5)	794.5777	-0.6293	2.19	(18:3/18:2), (20:4/16:1), (20:5/16:0) and (18:4/18:1)	C45H80NO10
MGDG(36:4)	796.5928	-0.6277	2.19	(18:2/18:2), (18:3/18:1) and (20:4/16:0)	C45H82NO10
MGDG(36:3)	798.6071	-3.0303	2.12	(18:2/18:1), (18:3/18:0), (16:0/20:3) and (16:1/20:2)	C45H84NO10
MGDG(36:2)	800.6249	-0.3422	2.31	(20:2/16:0), (18:0/18:2), (18:1/18:1) and (16:1/20:1)	C45H86NO10
MGDG(36:1)	802.6408	-0.0299	2.18	(20:1/16:0) and (18:0/18:1)	C45H88NO10
**MGDG(38:9)**	**814.5466**	**-0.3978**	**2.20**	**(20:5/18:4)**	**C47H76NO10**
MGDG(38:8)	816.5597	-3.5147	2.31	(20:4/18:4) and (20:5/18:3)	C47H78NO10
MGDG(38:7)	818.5759	-2.8391	2.31	(18:3/20:4) and (18:2/20:5)	C47H80NO10
MGDG(38:6)	820.5939	0.0000	2.20	(18:1/20:5)	C47H82NO10
MGDG(38:5)	822.6095	-0.0243	2.31	(18:2/20:3), (20:4/18:1), (20:2/18:3) and (20:1/18:4)	C47H84NO10
MGDG(38:4)	824.6251	0.0000	2.20	*	C47H86NO10
MGDG(40:10)	840.5637	1.3443	2.12	(20:5/20:5)	C49H78NO10
MGDG(40:9)	842.5781	-0.1187	2.20	(20:5/20:4)	C49H80NO10
MGDG(40:8)	844.5925	-1.6221	2.31	(20:4/20:4)	C49H82NO10
MGDG(40:6)	848.6252	0.0306	2.27	*	C49H86NO10

MGDG(42:9)	870.6088	-0.8316	1.67	*	C51H84NO10
			**MGMG identified as [M + NH_4]$^+$**		
MGMG(16:4)	502.3016	-0.0179	2.31	(16:4)	C25H44NO9
MGMG(16:3)	504.3173	0.0813	2.36	(16:3)	C25H46NO9
MGMG(16:1)	508.3485	-0.1161	2.31	(16:1)	C25H50NO9
MGMG(16:0)	510.3642	0.0000	2.36	(16:0)	C25H52NO9
**MGMG(18:4)**	**530.3326**	**-0.5827**	**2.38**	**(18:4)**	**C27H48NO9**
MGMG(18:3)	532.3487	0.2649	2.36	*	C27H50NO9
MGMG 18:2)	534.3641	-0.2040	2.36	*	C27H52NO9
MGMG(18:1)	536.3799	0.0764	2.31	(18:1)	C27H54NO9
MGMG(20:5)	556.3486	0.0737	2.36	(20:5)	C29H50NO9
MGMG(20:4)	558.3642	-0.0161	2.31	(20:4)	C29H52NO9
			**DGDG identified as [M + NH_4]$^+$**		
DGDG(28:0)	854.5827	-1.6382	2.12	(16:0/12:0)	C43H84O15N
DGDG(32:3)	904.5997	0.0000	2.38	*	C47H86O15N
DGDG(32:2)	906.6154	0.0000	2.36	(14:0/18:2)	C47H88O15N
DGDG(32:1)	908.6310	0.0000	2.31	(18:1/14:0) and (16:0/16:1)	C47H90O15N
DGDG(34:5)	928.5997	0.0000	2.36	*	C49H86O15N
DGDG(34:4)	930.6154	0.0000	2.38	(18:3/16:1) and (16:0/18:4)	C49H88O15N
DGDG(34:3)	932.6310	0.0000	2.38	(18:3/16:0) and (16:1/18:2)	C49H90O15N
DGDG(34:2)	934.6447	-2.1398	2.31	(18:2/16:0)	C49H92O15N
DGDG(34:1)	936.6608	-1.6014	2.43	(18:1/16:0)	C49H94O15N
DGDG(36:9)	948.5684	0.0000	2.36	*	C51H82O15N
DGDG(36:8)	950.5844	0.3156	2.38	(18:4/18:4)	C51H84O15N
DGDG(36:7)	952.6000	0.3149	2.04	(18:4/18:3) and (20:5/16:2)	C51H86O15N
DGDG(36:6)	954.6151	-0.3143	2.40	(18:3/18:3) and (20:5/16:1)	C51H88O15N
DGDG(36:5)	956.6307	-0.3136	2.38	(20:5/16:0) and (16:1/20:4)	C51H90O15N
DGDG(36:3)	960.6623	0.0000	2.40	*	C51H94O15N
DGDG(36:2)	962.6780	0.0000	2.40	*	C51H96O15N
**DGDG(38:9)**	**976.5997**	**0.0000**	**2.31**	**(20:5/18:4)**	**C53H86O15N**
			**SQDG identified as [M – H]$^-$**		
SQDG(28:0)	737.4517	0.9804	1.84	**	C37H69O12S
SQDG(30:1)	763.4666	-0.0354	1.76	(14:0/16:1)	C39H71O12S
SQDG(30:0)	765.4817	-0.7538	1.71	(14:0/16:0)	C39H73O12S
SQDG(32:4)	785.4509	-0.0980	1.72	*	C41H69O12S
SQDG(32:3)	787.4658	-1.0502	1.76	**	C41H71O12S
SQDG(32:2)	789.4819	-0.4775	1.64	(14:0/18:2), (16:1/16:1) and (16:0/16:2)	C41H73O12S
SQDG(32:1)	791.4996	2.1137	1.83	(16:1/16:0) and (14:0/18:1)	C41H75O12S
SQDG(32:0)	793.5146	1.2905	1.83	(16:0/16:0)	C41H77O12S
SQDG(34:5)	811.4666	-0.0333	1.71	*	C43H71O12S
SQDG(34:4)	813.4832	1.1346	1.74	(16:1/18:3) and (16:0/18:4)	C43H73O12S
SQDG(34:3)	815.4999	2.4194	1.64	(18:2/16:1) and (16:0/18:3)	C43H75O12S
SQDG(34:2)	817.5142	0.7633	1.71	(16:0/18:2) and (18:1/16:1)	C43H77O12S

**SQDG(34:1)**	**819.5292**	**-0.0329**	**1.76**	**(16:0/18:1)**	**C43H79O12S**
SQDG(36:7)	835.4669	0.3268	1.72	**	C45H71O12S
SQDG(36:6)	837.4821	-0.2113	1.76	(18:3/18:3)	C45H73O12S
SQDG(36:5)	839.4995	1.8737	1.64	(20:5/16:0), (20:4/16:1) and (18:3/18:2)	C45H75O12S
SQDG(36:4)	841.5121	-1.7540	1.76	(20:4/16:0) and (18:4/18:0)	C45H77O12S
SQDG(36:3)	843.5291	-0.1506	1.74	*	C45H79O12S
SQDG(36:2)	845.5428	-2.4564	1.76	(20:2/16:0)	C45H81O12S
SQDG(36:1)	847.5624	2.2099	1.83	(20:1/16:0)	C45H83O12S
SQDG(38:9)	859.4667	0.0849	1.74	**	C47H71O12S
SQDG(38:8)	861.4819	-0.4376	1.73	**	C47H73O12S
SQDG(38:7)	863.4980	0.0845	1.74	**	C47H75O12S
SQDG(38:6)	865.5121	-1.7053	1.70	**	C47H77O12S
SQDG(38:5)	867.5299	0.7758	1.70	**	C47H79O12S
SQDG(38:2)	873.5769	0.8276	1.83	**	C47H85O12S
SQDG(38:1)	875.5903	-1.7440	1.71	(22:1/16:0)	C47H87O12S
SQDG(38:0)	877.6070	-0.5435	1.71	(22:0/16:0)	C47H89O12S
SQDG(40:8)	889.5137	0.1394	1.73	(20:4/20:4)	C49H77O12S
SQDG(40:6)	893.5448	-0.0862	1.71	*	C49H81O12S
SQDG(40:1)	903.6245	1.5194	1.74	**	C49H91O12S
SQDG(40:0)	905.6388	0.0254	1.71	*	C49H93O12S
**LPC identified as [M + H]+**					
**LPC(16:0)**	**496.3403**	**-0.0322**	**15.07**	**	**C24H51NO7P**
LPC(18:1)	522.3560	0.0632	15.07	**	C26H53NO7P
LPC(18:0)	524.3716	-0.0324	14.92	*	C26H55NO7P
**PC identified as [M + H]+**					
**PC(30:3)**	**700.4896**	**-3.0436**	**11.73**	*	**C38H71NO8P**
PC(34:2)	758.5685	-1.9537	12.19	**	C42H81NO8P
PC(34:1)	760.5848	-1.0939	12.33	**	C42H83NO8P
PC(36:5)	780.5541	-0.2959	11.89	*	C44H79NO8P
PC(36:4)	782.5709	1.1731	10.98	**	C44H81NO8P
PC(36:2)	786.6012	-0.1042	11.84	*	C44H85NO8P
PC(36:1)	788.6165	-0.5478	11.34	*	C44H87NO8P
PC(38:7)	804.5543	-0.0385	10.88	*	C46H79NO8P
PC(38:6)	806.5696	-0.4736	11.10	**	C46H81NO8P
PC(40:10)	826.5385	-0.2202	10.88	*	C48H77NO8P
**LPE identified as [M + H]+**					
LPE(20:5)	500.2778	0.1659	5.95	**	C25H43NO7P
**LPE(20:4)**	**502.2926**	**-1.5270**	**5.76**	**(20:4)**	**C25H45NO7P**
**PE identified as [M + H]+**					
PE(30:3)	658.4425	-3.4657	4.60	**	C35H65NO8P
PE(30:1)	662.4761	0.0272	4.41	*	C35H69NO8P
PE(32:2)	688.4927	1.4060	4.33	*	C37H71O8NP
PE(32:1)	690.5068	-0.8429	4.41	(16:1/16:0)	C37H73NO8P

PE(34:5)	710.4758	-0.3969	4.04	*	C39H69NO8P
PE(34:4)	712.4918	0.0954	4.10	(20:4/14:0)	C39H71NO8P
PE(34:3)	714.5074	0.0252	4.04	*	C39H73O8NP
PE(34:2)	716.5230	-0.0447	4.10	**	C39H75NO8P
PE(34:1)	718.5385	-0.2533	4.08	(14:0/20:1)	C39H77NO8P
PE(36:7)	734.4758	-0.3839	4.08	**	C41H69NO8P
PE(36:6)	736.4922	0.6354	4.04	**	C41H71NO8P
PE(36:5)	738.5068	-0.7881	4.10	(20:5/16:0) and (16:1/20:4)	C41H73O8NP
PE(36:4)	740.5230	-0.0432	4.32	**	C41H75NO8P
PE(38:9)	758.4744	-2.2176	4.13	*	C43H69NO8P
PE(38:8)	760.4890	-3.5924	3.97	(18:4/20:4)	C43H71NO8P
PE(38:7)	762.5063	-1.4190	3.97	(18:2/20:5) and (20:4/18:3)	C43H73NO8P
PE(38:6)	764.5227	-0.4343	4.13	(20:4/18:2)	C43H75O8NP
PE(38:5)	766.5370	-2.1943	3.97	(20:4/18:1) and (20:5/18:0)	C43H77NO8P
PE(38:4)	768.5536	-0.9511	3.97	**	C43H79O8NP
PE(38:3)	770.5700	0.0234	3.97	**	C43H81NO8P
PE(40:10)	784.4921	0.4691	3.97	(20:5/20:5)	C45H71NO8P
PE(40:9)	786.5071	-0.3585	3.73	(20:4/20:5)	C45H73O8NP
**PE(40:8)**	**788.5226**	**-0.5479**	**3.73**	**(20:4/20:4) and (20:3/20:5)**	**C45H75O8NP**
PE(40:7)	790.5395	1.0347	3.93	(20:5/20:2) and (20:4/20:3)	C45H77NO8P
PE(40:6)	792.5547	0.4656	3.93	(20:4/20:2) and (20:1/20:5)	C45H79O8NP
PE(40:5)	794.5705	0.6519	3.73	(20:5/20:0), (20:4/20:1) and (20:2/20:3)	C45H81O8NP
PE(40:4)	796.5858	0.2109	4.07	(20:4/20:0)	C45H83NO8P
PE(42:11)	810.5053	-2.5688	4.13	**	C47H73NO8P
PE(42:7)	818.5700	0.0220	3.93	**	C47H81O8NP
PE(42:6)	820.5873	2.0327	4.07	(20:5/22:1) and (20:4/22:2)	C47H83NO8P
PE(42:5)	822.6011	-0.2212	3.81	(20:4/22:1) and (20:5/22:0)	C47H85O8NP
PE(42:4)	824.6169	-0.0388	3.81	**	C47H87O8NP
PE(44:7)	846.6012	-0.0969	4.07	(20:4/24:3)	C49H85NO8P
PE(44:4)	852.6482	-0.0375	3.87	**	C49H91NO8P
			**LPG identified as [M – H]⁻**		
**LPG(16:0)**	**483.2733**	**2.0734**	**2.16**	**(16:0)**	**C22H44O9P**
			**PG identified as [M – H]⁻**		
PG(30:0)	693.4733	3.7493	2.14	*	C36H70O10P
PG(32:2)	717.4707	0.0000	1.93	(16:1/16:1) and (16:0/16:2)	C38H70O10P
PG(32:1)	719.4867	0.5560	1.90	(16:1/16:0)	C38H72O10P
PG(32:0)	721.5020	0.0000	2.02	(16:0/16:0)	C38H74O10P
PG(34:5)	739.4550	0.0000	1.91	(18:4/16:1) and (16:0/18:5)	C40H68O10P
**PG(34:4)**	**741.4710**	**0.4046**	**1.90**	**(18:3/16:1)**	**C40H70O10P**
PG(34:3)	743.4863	0.0000	1.95	(16:0/18:3) and (18:2/16:1)	C40H72O10P
PG(34:2)	745.5016	-0.5366	1.93	(14:0/20:2), (16:1/18:1) and (16:0/18:2)	C40H74O10P
PG(34:1)	747.5179	0.4013	1.91	(14:0/20:1), (16:1/18:0) and (18:1/16:0)	C40H76O10P
PG(36:4)	769.5020	0.0000	1.91	(20:4/16:0), (18:2/18:2) and (18:3/18:1)	C42H74O10P

PG(36:3)	771.5176	0.0000	1.98	(18:2/18:1), (16:1/20:2) and (16:0/20:3)	C42H76O10P
PG(36:2)	773.5333	0.0000	1.91	(20:1/16:1), (16:0/20:2) and (18:1/18:1)	C42H78O10P
PG(38:5)	795.5176	0.0000	1.71	(20:4/18:1) and (20:0/18:5)	C44H76O10P
PG(40:6)	821.5357	2.9214	1.73	*	C46H78O10P
PG(42:6)	849.5670	2.8250	1.71	(20:4/22:2), (18:4/24:2), (18:3/24:3) and (20:5/22:1)	C48H82O10P
**PI identified as [M – H]⁻**					
PI(28:2)	749.4241	0.0000	2.24	*	C37H66O13P
PI(28:1)	751.4398	0.0000	2.18	*	C37H68O13P
**PI(34:2)**	**833.5180**	**0.0000**	**2.12**	**(18:2/16:0)**	**C43H78O13P**
PI(34:1)	835.5337	0.0000	2.07	(16:0/18:1)	C43H80O13P
PI(38:10)	873.4554	-0.0092	1.72	*	C47H70O13P
PI(38:8)	877.4867	0.0000	1.84	*	C47H74O13P
PI(42:11)	927.5047	2.4798	2.10	*	C51H76O13P
PI(42:8)	933.5493	0.0000	1.77	*	C51H82O13P
**DGTS identified as [M + H]⁺**					
DGTS(30:1)	682.5620	-0.2930	4.34	*	C40H76O7N
DGTS(32:2)	708.5779	0.1411	4.30	(18:2/14:0)	C42H78O7N
DGTS(32:1)	710.5913	-3.0960	4.34	(16:1/16:0)	C42H80O7N
DGTS(34:5)	730.5622	0.0000	4.38	*	C44H76O7N
DGTS(34:4)	732.5760	-2.4571	4.33	*	C44H78O7N
DGTS(34:3)	734.5915	-2.7226	4.30	*	C44H80O7N
**DGTS(34:2)**	**736.6100**	**1.2218**	**4.30**	**(16:0/18:2)**	**C44H82O7N**
DGTS(34:1)	738.6245	-0.4062	4.08	(16:0/18:1)	C44H84O7N
DGTS(36:7)	754.5622	0.0000	4.10	*	C46H76O7N
DGTS(36:6)	756.5769	-1.1896	4.10	*	C46H78O7N
DGTS(36:5)	758.5932	-0.3955	4.17	*	C46H80O7N
DGTS(36:4)	760.6083	-1.0518	4.13	*	C46H82O7N
DGTS(38:5)	786.6241	-0.8899	4.32	(18:1/20:4)	C48H84O7N
**DGTA identified as [M + H]⁺**					
DGTA(28:1)	654.5309	0.0000	9.55	*	C38H72O7N
DGTA(28:0)	656.5466	0.1523	10.29	*	C38H74O7N
DGTA(30:3)	678.5309	0.0000	9.95	*	C40H72O7N
DGTA(30:2)	680.5444	-3.0858	10.29	*	C40H74O7N
DGTA(30:1)	682.5610	-1.7581	10.43	*	C40H76O7N
DGTA(30:0)	684.5778	0.0000	10.29	*	C40H78O7N
DGTA(32:4)	704.5446	-2.6968	10.06	(16:3/16:1)	C42H74O7N
DGTA(32:3)	706.5618	-0.5661	10.52	(14:0/18:3)	C42H76O7N
DGTA(32:2)	708.5780	0.2823	10.70	(16:0/16:2) and (18:2/14:0)	C42H78O7N
DGTA(32:1)	710.5916	-2.6738	10.41	(16:1/16:0)	C42H80O7N
DGTA(34:6)	728.5443	-3.0197	10.09	*	C44H74O7N
DGTA(34:5)	730.5622	0.0000	9.70	*	C44H76O7N
DGTA(34:4)	732.5757	-2.8666	9.35	(20:4/14:0)	C44H78O7N
DGTA(34:3)	734.5916	-2.5865	9.70	(16:0/18:3)	C44H80O7N

DGTA(34:2)	736.6071	-2.7151	9.43	(18:2/16:0)	C44H82O7N
DGTA(34:1)	738.6248	0.0000	9.70	(16:0/18:1)	C44H84O7N
DGTA(36:8)	752.5461	-0.5581	9.70	*	C46H74O7N
DGTA(36:7)	754.5621	-0.1325	9.70	**	C46H76O7N
DGTA(36:6)	756.5762	-2.1148	9.70	*	C46H78O7N
DGTA(36:5)	758.5930	-0.6591	9.70	(18:2/18:3), (20:5/16:0). and (20:4/16:1)	C46H80O7N
**DGTA(36:4)**	**760.6084**	**-0.9203**	**9.29**	**(16:0/20:4)**	**C46H82O7N**
DGTA(36:2)	764.6391	-1.7001	9.70	(20:2/16:0)	C46H86O7N
DGTA(36:1)	766.6561	0.0000	9.34	(16:0/20:1)	C46H88O7N
DGTA(38:8)	780.5751	-3.4590	9.29	*	C48H78O7N
DGTA(38:7)	782.5914	-2.6834	9.34	(18:3/20:4)	C48H80O7N
DGTA(38:6)	784.6084	-0.8922	9.29	(18:2/20:4)	C48H82O7N
DGTA(38:5)	786.6235	-1.6526	9.70	(20:4/18:1)	C48H84O7N
DGTA(38:4)	788.6402	-0.2536	9.34	*	C48H86O7N
DGTA(40:10)	804.5759	-2.3615	9.29	*	C50H78O7N
DGTA(40:9)	806.5915	-2.4796	9.70	(20:4/20:5)	C50H80O7N
DGTA(40:8)	808.6091	0.0000	9.34	(20:4/20:4)	C50H82O7N
DGTA(40:6)	812.6392	-1.4767	9.36	(20:2/20:4)	C50H86O7N
DGTA(40:5)	814.6561	0.0000	9.34	(20:1/20:4)	C50H88O7N
DGTA(42:11)	830.5935	0.0253	9.34	*	C52H80O7N
DGTA(42:9)	834.6220	-3.3296	9.29	*	C52H84O7N
DGTA(42:6)	840.6731	1.6308	8.88	*	C52H90O7N
DGTA(42:5)	842.6885	1.3303	8.58	*	C52H92O7N

*: Molecular species identified by retention time and mass accuracy;

**: Molecular species identified by retention time, mass accuracy, and polar head product ion;

#: Contribution of sodiated adduct ion

Table S3 List of common and unique lipid species in the lipidome of *B. bifurcata* and *S. muticum*.

Unique lipid species		Common lipid species
Bifurcaria bifurcata	*Sargassum muticum*	*B. bifurcata*∣*S. muticum*
MGDG(30:4)	MGDG(30:0)	MGDG(30:1)
MGDG(38:10)	MGDG(32:5)	MGDG(32:1)
DGDG(36:4)	MGDG(32:8)	MGDG(32:2)
DGDG(38:8)	MGDG(34:5)	MGDG(32:3)
SQDG(34:0)	MGDG(34:6)	MGDG(32:4)
SQDG(38:4)	MGDG(34:7)	MGDG(34:1)
SQDG(40:7)	MGDG(36:1)	MGDG(34:2)
PE(36:2)	MGDG(36:9)	MGDG(34:3)
PE(36:3)	MGDG(38:4)	MGDG(34:4)
PG(34:0)	MGDG(40:6)	MGDG(36:2)
PG(36:5)	MGDG(42:9)	MGDG(36:3)
PI(30:2)	MGMG(16:0)	MGDG(36:4)
PI(36:8)	MGMG(16:1)	MGDG(36:5)
PI(40:5)	MGMG(16:3)	MGDG(36:6)
PI(40:6)	MGMG(16:4)	MGDG(36:7)
PI(46:3)	MGMG(18:1)	MGDG(36:8)
DGTA(36:3)	MGMG(18:2)	MGDG(38:5)
DGTA(40:7)	MGMG(18:3)	MGDG(38:6)
DGTA(42:8)	MGMG(18:4)	MGDG(38:7)
	MGMG(20:4)	MGDG(38:8)
	MGMG(20:5)	MGDG(38:9)
	DGDG(34:5)	MGDG(40:10)
	DGDG(36:2)	MGDG(40:8)
	DGDG(36:3)	MGDG(40:9)
	DGDG(36:8)	DGDG(28:0)
	DGDG(36:9)	DGDG(32:1)
	SQDG(28:0)	DGDG(32:2)
	SQDG(32:4)	DGDG(32:3)
	SQDG(36:1)	DGDG(34:1)
	SQDG(38:1)	DGDG(34:2)
	SQDG(38:2)	DGDG(34:3)
	SQDG(40:1)	DGDG(34:4)
	SQDG(40:6)	DGDG(36:5)
	LPC(16:0)	DGDG(36:6)
	LPC(18:0)	DGDG(36:7)
	LPC(18:1)	DGDG(38:9)
	PC(34:1)	SQDG(30:0)
	PC(36:1)	SQDG(30:1)
	PC(36:2)	SQDG(32:0)
	PC(36:4)	SQDG(32:1)
	PC(36:5)	SQDG(32:2)
	PC(38:7)	SQDG(32:3)
	PC(40:10)	SQDG(34:1)
	LPE(20:4)	SQDG(34:2)
	LPE(20:5)	SQDG(34:3)
	PE(30:1)	SQDG(34:4)
	PE(32:1)	SQDG(34:5)
	PE(32:2)	SQDG(36:2)
	PE(34:3)	SQDG(36:3)
	PE(34:5)	SQDG(36:4)
	PE(36:6)	SQDG(36:5)
	PE(36:7)	SQDG(36:6)
	PE(38:3)	SQDG(36:7)
	PE(38:9)	SQDG(38:0)
	PE(42:6)	SQDG(38:5)

	PE(42:7)	SQDG(38:6)
	PE(44:7)	SQDG(38:7)
	LPG(16:0)	SQDG(38:8)
	PG(30:0)	SQDG(38:9)
	PG(32:0)	SQDG(40:0)
	PG(32:2)	SQDG(40:8)
	PG(34:5)	PC(30:3)
	PG(38:5)	PC(34:2)
	PG(40:6)	PC(38:6)
	PG(42:6)	PE(30:3)
	PI(28:1)	PE(34:1)
	PI(28:2)	PE(34:2)
	PI(34:2)	PE(34:4)
	PI(38:10)	PE(36:4)
	PI(42:11)	PE(36:5)
	PI(42:8)	PE(38:4)
	DGTS(30:1)	PE(38:5)
	DGTS(34:3)	PE(38:6)
	DGTS(34:4)	PE(38:7)
	DGTS(34:5)	PE(38:8)
	DGTS(36:5)	PE(40:10)
	DGTS(36:6)	PE(40:4)
	DGTS(36:7)	PE(40:5)
	DGTS(38:5)	PE(40:6)
	DGTA(28:0)	PE(40:7)
	DGTA(28:1)	PE(40:8)
	DGTA(30:0)	PE(40:9)
	DGTA(30:2)	PE(42:11)
	DGTA(30:3)	PE(42:4)
	DGTA(34:6)	PE(42:5)
	DGTA(36:1)	PE(44:4)
	DGTA(36:8)	PG(32:1)
	DGTA(38:4)	PG(34:1)
	DGTA(40:10)	PG(34:2)
	DGTA(40:5)	PG(34:3)
	DGTA(42:5)	PG(34:4)
	DGTA(42:6)	PG(36:2)
	DGTA(42:9)	PG(36:3)
		PG(36:4)
		PI(34:1)
		PI(38:8)
		DGTS(32:1)
		DGTS(32:2)
		DGTS(34:2)
		DGTS(34:1)
		DGTS(36:4)
		DGTA(30:1)
		DGTA(32:1)
		DGTA(32:2)
		DGTA(32:3)
		DGTA(32:4)
		DGTA(34:1)
		DGTA(34:2)
		DGTA(34:3)
		DGTA(34:4)
		DGTA(34:5)
		DGTA(36:2)
		DGTA(36:4)
		DGTA(36:5)
		DGTA(36:6)
		DGTA(36:7)

	DGTA(38:5)
	DGTA(38:6)
	DGTA(38:7)
	DGTA(38:8)
	DGTA(40:6)
	DGTA(40:8)
	DGTA(40:9)
	DGTA(42:11)
